# Supplementary material for: Deletion of APC7 or APC16 Allows Proliferation of Human Cells without the Spindle Assembly Checkpoint
Source: Cell Rep. 2018 Nov 27;25(9):2317–2328.e5. doi: 10.1016/j.celrep.2018.10.104 (PMC6289045; doi:10.1016/j.celrep.2018.10.104)
Supplement: Document S1. Figures S1–S5 [file mmc1.pdf]

Cell Reports, Volume *25*

Supplemental Information

Deletion of APC7 or APC16 Allows Proliferation of Human Cells without the Spindle Assembly Checkpoint

Thomas Wild, Magda Budzowska, Susanne Hellmuth, Susana Eibes, Gopal Karemore, Marin Barisic, Olaf Stemmann, and Chunaram Choudhary

Figure S1

A

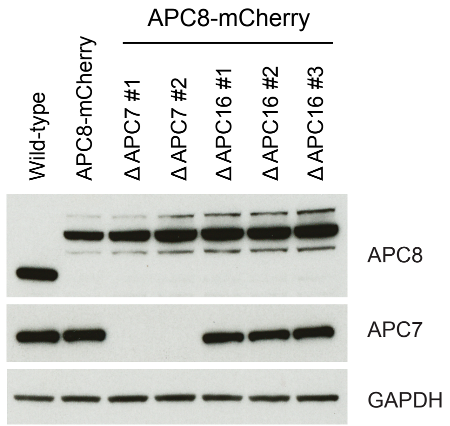

B

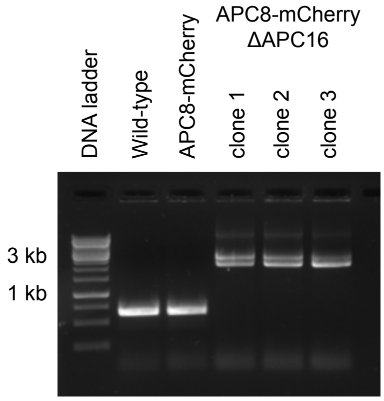

C

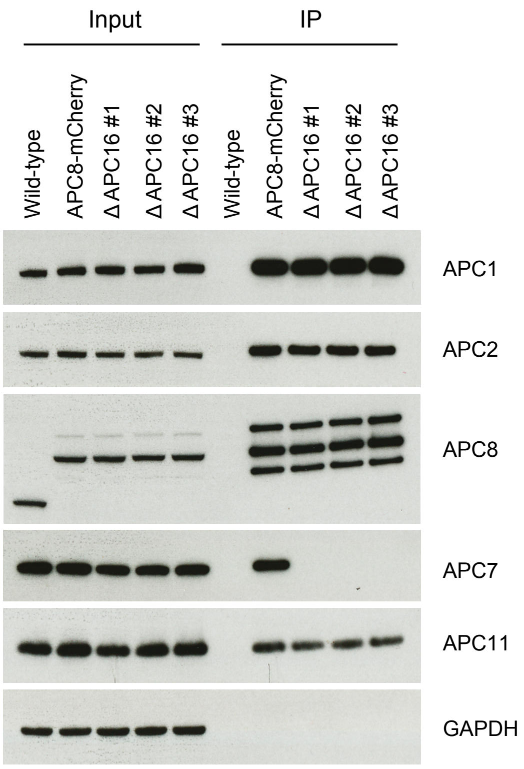

D

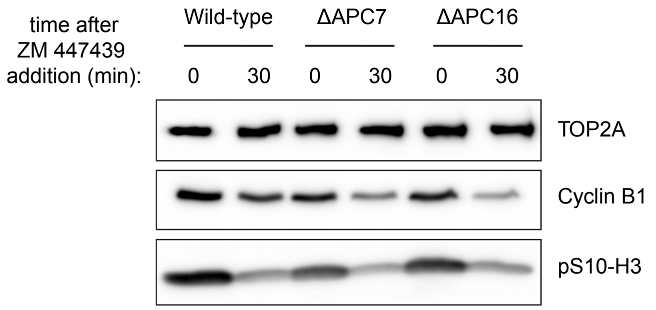

E

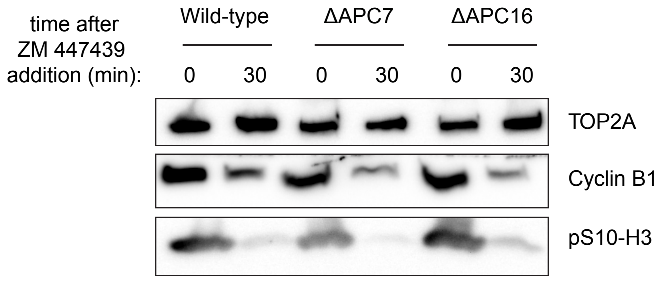

Figure S1. Related to Figure 1 and 2.

A) Generation of APC8-mCherry, ∆APC7 and ∆APC16 cell lines to analyse APC/C composition. ∆APC7 and ∆APC16 cells were generated in the APC8-mCherry background. Immunoblot analysis of wild-type, APC8-mCherry, APC8-mCherry ∆APC7 and APC8-mCherry ∆APC16 cell lines using the indicated antibodies. Two independent ∆APC7 and three independent ∆APC16 clonal cell lines are shown. GAPDH levels were analysed as a loading control.

B) Genomic APC16 locus from wild-type, APC8-mCherry and APC8-mCherry ∆APC16 cells analysed by PCR. The used primer pair amplifies an approximately 600 base pair (bp) product from a wild-type APC16 locus and an approximately 2300 or 2900 bp product upon genomic insertion of the deletion cassette. Note that biallelic disruption of the APC16 locus was performed using two deletion cassettes bearing different selection markers, which results in two differently sized PCR products. Three independent ∆APC16 clonal cell lines are shown.

C) Analysis of APC/C composition in ∆APC16 cells. APC/C was purified via APC8-mCherry pulldowns from APC8-mCherry and APC8-mCherry ∆APC16 cells (from three independent ∆APC16 clonal cell lines) and subsequently analyzed by immunoblotting using the indicated antibodies. Wild-type cells served as a control for unspecific binding to the affinity beads. GAPDH levels were analysed to verify equal amount of input for the different cell lines.

D) Immunoblot analysis of the indicated cell lines after the release from the prometaphase arrest used for Figure 2A. 30 min after release from taxol into ZM containing medium, cells were harvested for APC/C purification. Cyclin B1 and phospho-H3 (Ser 10) levels were assessed to compare mitotic progression, TOP2A levels were analysed as a loading control. ZM = ZM 447439.

E) Immunoblot analysis of the indicated cell lines after the release from the prometaphase arrest, used in Figure 2C, analysed as described in D).

Figure S2

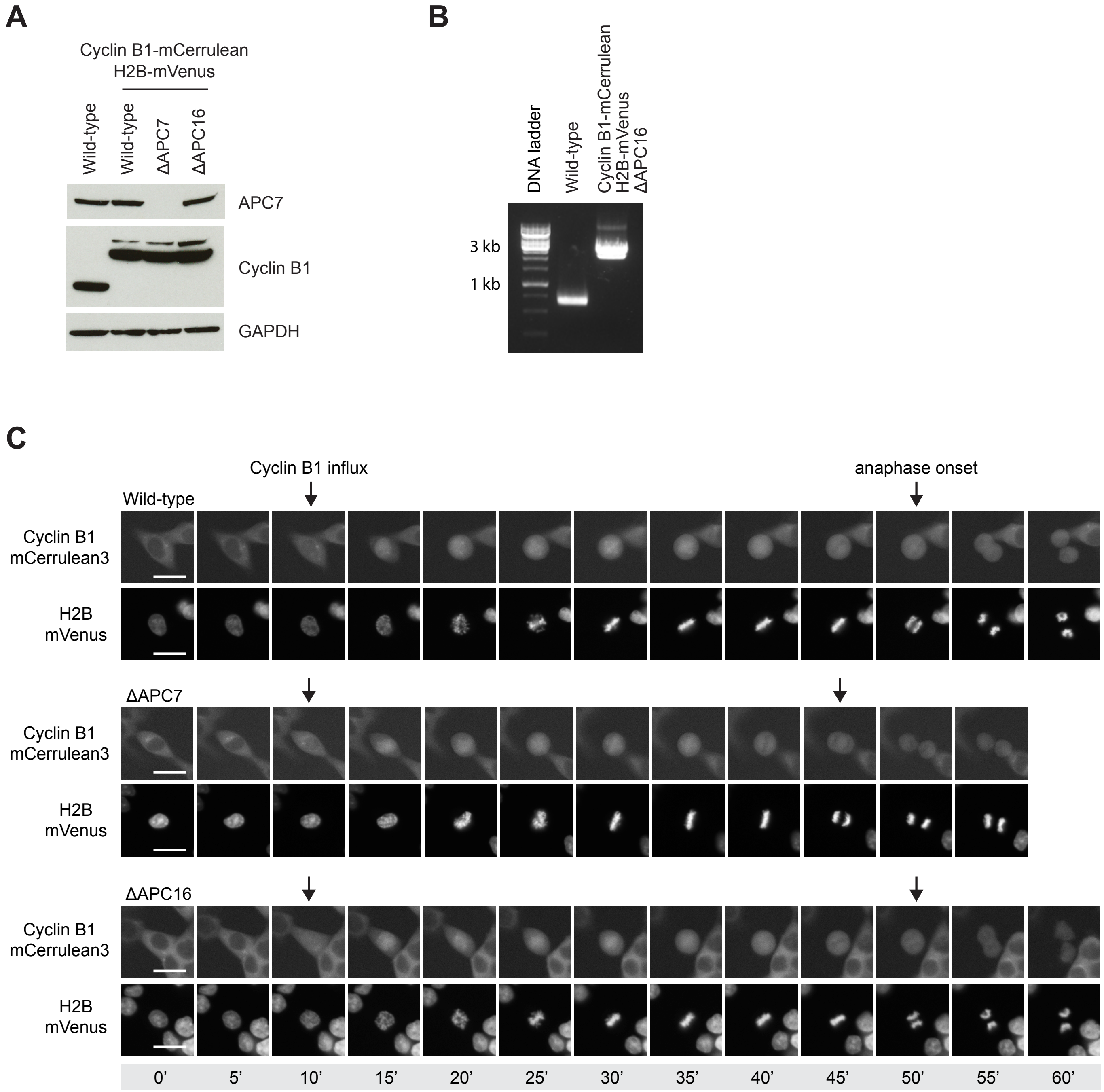
A
Cyclin B1-mCerrulean
H2B-mVenus
Wild-type
Wild-type
ΔAPC7
ΔAPC16
APC7
Cyclin B1
GAPDH
B
DNA ladder
Wild-type
Cyclin B1-mCerrulean
H2B-mVenus
ΔAPC16
3 kb
1 kb
C
Cyclin B1 influx
anaphase onset
Wild-type
Cyclin B1
mCerrulean3
H2B
mVenus
ΔAPC7
Cyclin B1
mCerrulean3
H2B
mVenus
ΔAPC16
Cyclin B1
mCerrulean3
H2B
mVenus
0'
5'
10'
15'
20'
25'
30'
35'
40'
45'
50'
55'
60'

Figure S2. Related to Figure 3.

A) Immunoblot analysis of the cell lines used for live cell imaging data shown in Figure 3A and 3B. Lysates from the indicated cell lines were separated by SDS-PAGE and analysed with the indicated antibodies. GAPDH levels were analysed to control for equal loading.

B) Genomic APC16 locus from wild-type and cyclin B1-mCerulean H2B-mVenus ΔAPC16 cells analysed by PCR as described in Figure S1B.

C) Representative images extracted from time-lapse imaging experiments shown in Figure 3A and Figure 3B. For the different cell lines, the cyclin B1 influx frame (cyclin B1-mCerulean3 channel) and anaphase onset frame (H2B-mVenus) are indicated as the first and second arrow, respectively. The scale bars indicate 10 μm. Time points (in minutes) are listed in the grey bar below the images.

Figure S3

A

noc (-)
noc (+)

Wild-type

	noc (-)	noc (+)
2N	53.6	1.9
2-4N	17.4	0.9
4N	26.0	93.4
>4N	2.3	3.5

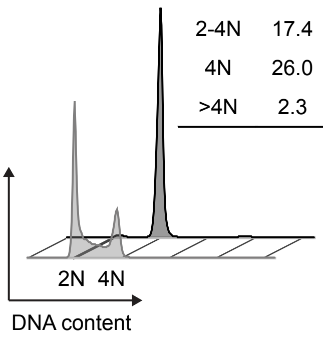

APC8-mCherry

	noc (-)	noc (+)
2N	57.5	1.8
2-4N	23.6	1.1
4N	16.4	94.0
>4N	1.7	2.9

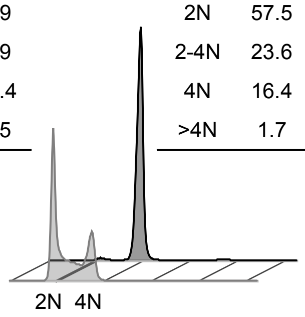

ΔAPC7

	noc (-)	noc (+)
2N	50.7	2.1
2-4N	19.6	1.9
4N	26.5	93.0
>4N	2.6	2.8

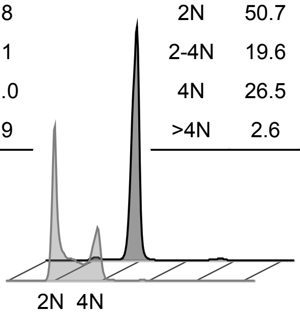

ΔAPC16

	noc (-)	noc (+)
2N	56.9	1.8
2-4N	19.5	2.1
4N	21.1	93.8
>4N	1.8	2.2

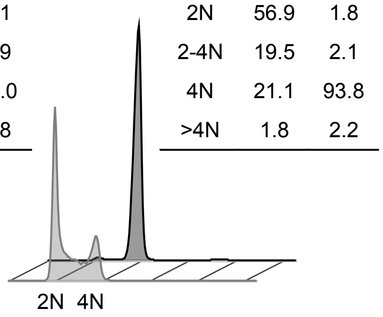

B

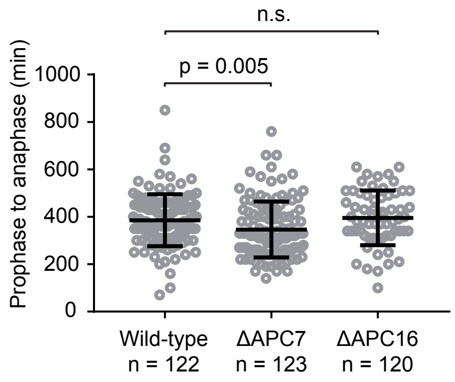

C

rev (-)
rev (48h)
rev (96h)

Wild-type

	2-4N	>4N
rev (-)	99.8 ± 0.1	0.2 ± 0.1
rev (48h)	60 ± 1.8	40 ± 1.8
rev (96h)	37.3 ± 1.2	62.7 ± 1.2

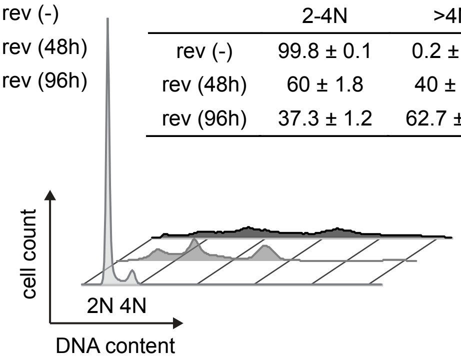

ΔAPC7

	2-4N	>4N
rev (-)	97.2 ± 0.4	2.8 ± 0.4
rev (48h)	53.3 ± 5.3	46.7 ± 5.3
rev (96h)	25.9 ± 2.7	74.1 ± 2.7

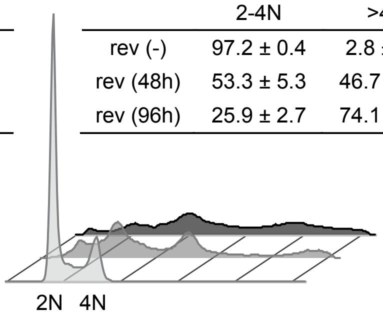

ΔAPC16

	2-4N	>4N
rev (-)	98.5 ± 0.6	1.5 ± 0.6
rev (48h)	76.8 ± 3.4	23.2 ± 3.4
rev (96h)	22.9 ± 0.5	77.1 ± 0.5

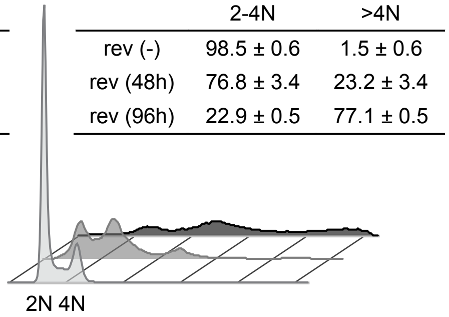

D

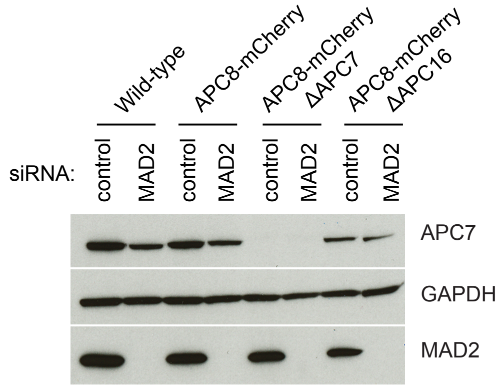

Figure S3. Related to Figure 3.

A) Analysis of cellular DNA content in wild-type, ΔAPC7 and ΔAPC16 cells upon 18 hours of nocodazole treatment. The DNA content in wild-type, ΔAPC7 and ΔAPC16 cells, grown with or without 18 hours of 200 nM nocodazole, was analyzed with propidium iodide staining and flow cytometry. The tables show the percentage of cells with the respective (2N, 2N-4N, 4N and >4N) DNA content.

B) Timing from prophase to anaphase for the indicated cell lines treated with 100 nM nocodazole. Nocodazole was added 2 hours before live cell imaging and for each cell line, the number of analysed cells is given as n. A two-tailed t-test was performed to calculate significance.

C) Analysis of cellular DNA content in wild-type, ΔAPC7 and ΔAPC16 cells upon 18 hours of nocodazole treatment. Analysis of DNA content in wild-type, ΔAPC7 and ΔAPC16 cells, grown with or without 18 hours of 200 nM nocodazole. Cells were stained with propidium iodide and analysed by flow cytometry. The tables show the percentage of cells with the respective (2N, 2N-4N, 4N and >4N) DNA content.

D) Immunoblot analysis of control and MAD2 RNAi used in Figure 3E-F. Cells imaged for Figure 3E and Figure 3F were lysed and expression of the indicated proteins was analysed by immunoblotting. A representative blot of the three independently performed experiments is shown. GAPDH levels were analysed to control for equal loading.

Figure S4

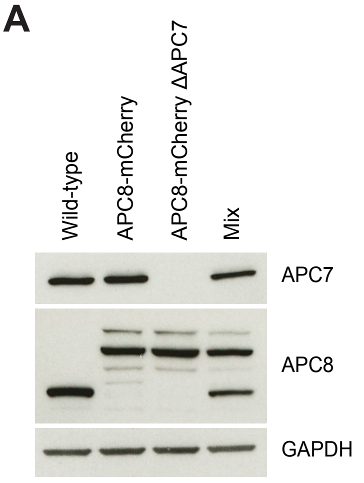

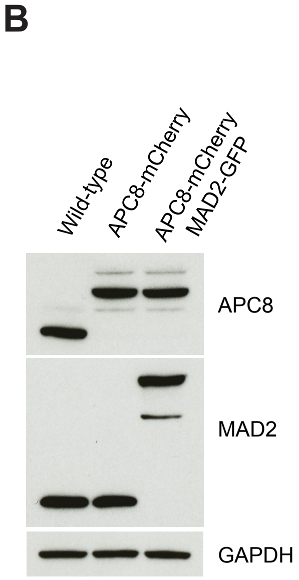

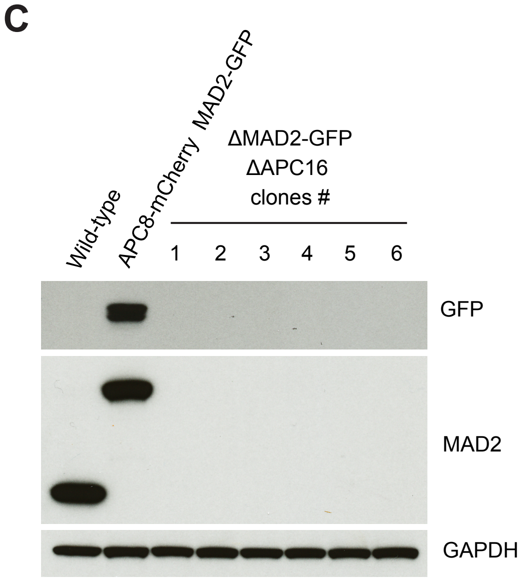

D

APC8-mCherry
ΔMAD2-GFP
ΔAPC16
clone #1

Allele 1:
ATGGCTGCTTCATCATCATCCTCCTCAGCTGGTGGGGTCAGTGGAAGTTCTGTCACTGGATCTGGTTTCAGT
GTCTCAGACCTTGCCCCACCACGGAAAGCCCTTTTCACCTACCCCAA**AGG**AGCTGGAGAGATGTTAGAAG

Allele 2:
ATGGCTGCTTCATCATCATCCTCCTCAGCTGGTGGGGTCAGTGGAAGTTCTGTCACTGGATCTGGTTTCAGT
GTCTCAGACCTTGCCCCACCACGGAAAGCCCTTTTCACCTACCCCAA**AGG**AGCTGGAGAGATGTTAGAAG

APC8mCherry
ΔMAD2-GFP
ΔAPC16
clone #2

Allele 1:
ATGGCTGCTTCATCATCATCCTCCTCAGCTGGTGGGGTCAGTGGAAGTTCTGTCACTGGATCTGGTTTCAGT
GTCTCAGACCTTGCCCCACCACGGAAAGCCCTTTTCACCTACCCCAA**AGG**AGCTGGAGAGATGTTAGAAG

Allele 2:
ATGGCTGCTTCATCATCATCCTCCTCAGCTGGTGGGGTCAGTGGAAGTTCTGTCACTGGATCTGGTTTCAGT
GTCTCAGACCTTGCCCCACCACGGAAAGCCCTTTTCACCTACCCCAA**AGG**AGCTGGAGAGATGTTAGAAG

APC8-mCherry
ΔMAD2-EGFP
ΔAPC16
clone #3

Allele 1:
ATGGCTGCTTCATCATCATCCTCCTCAGCTGGTGGGGTCAGTGGAAGTTCTGTCACTGGATCTGGTTTCAGT
GTCTCAGACCTTGCCCCACCACGGAAAGCCCTTTTCACCTACCCCAA**AGG**AGCTGGAGAGATGTTAGAAG

Allele 2:
ATGGCTGCTTCATCATCATCCTCCTCAGCTGGTGGGGTCAGTGGAAGTTCTGTCACTGGATCTGGTTTCAGTGT
CTCAGACCTTGCCCCACCACGGAAAGCCCTTTTCACCTACCCCAA**AGG**AGCTGGAGAGATGTTAGAAG

APC8-mCherry
ΔMAD2-GFP
ΔAPC16
clone #4

Allele 1:
ATGGCTGCTTCATCATCATCCTCCTCAGCTGGTGGGGTCAGTGGAAGTTCTGTCACTGGATCTGGTTTCAGTGT
CTCAGACCTTGCCCCACCACGGAAAGCCCTTTTCACCTACCCCAA**AGG**AGCTGGAGAGATGTTAGAAG

Allele 2:
ATGGCTGCTTCATCATCATCCTCCTCAGCTGGTGGGGTCAGTGGAAGTTCTGTCACTGGATCTGGTTTCAGTGT
CTCAGACCTTGCCCCACCACGGAAAGCCCTTTTCACCTACCCAGTATAATCTGCAGTCAATATTTCTGCGAAAGC
TTACTACTTCAAAGCCTTAAAAAGTGCATACTTTGGTCCAGCAAGTACCATTCCAATAATTTTTCTGAGAAAATAAT
CAAGGATACCAA**AGG**AGCTGGAGAGATGTTAGAAG

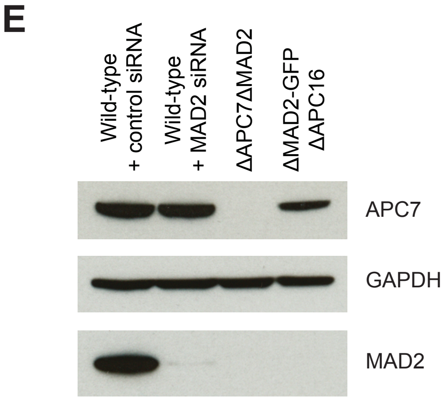

Figure S4. Related to Figure 4.

A) Immunoblot of cells used for mixing experiment outlined in Figure 4A. Equal number of wild-type, APC8-mCherry and APC8-mCherry ∆APC7 cells were mixed and seeded together. Lysate from the individual cell lines and the resulting mixed cell population (on the day of transfection of MAD2-targeting Crispr/Cas9 plasmids) were analysed for expression of indicated proteins with immunoblotting.

B) Immunoblot analysis of the APC8-mCherry MAD2-GFP cell line along with its parental cell lines with the indicated antibodies.

C) Immunoblot analysis of MAD2 expression in the six individual clones retrieved from MAD2-GFP synthetic viability assay performed in combination with an APC16 targeting guide RNA. Immunoblot for GAPDH serves as loading control.

D) Confirmation of APC16 locus disruption in four ∆MAD2-GFP∆APC16 clones. The genomic APC16 locus surrounding the guide RNA target site was cloned from four different ∆MAD2-GFP∆APC16 clonal cell lines and sequenced. The table lists the obtained sequences, beginning with the start codon of APC16 and depicting the PAM sequence adjacent to the guide RNA in bold. Compared to the wild-type APC16 locus, deleted base pairs are shown in boxes, inserted base pairs are highlighted with grey background. Note that all sequenced mutations cause disruption of the APC16 reading frame.

E) Immunoblot analysis of ∆APC7∆MAD2, ∆MAD2-GFP∆APC16 and MAD2 RNAi in wild-type HCT116 cells, used in Figure 4E. A representative blot of the three independently performed experiments is shown. GAPDH levels were analysed to control for equal loading.

Figure S5

A

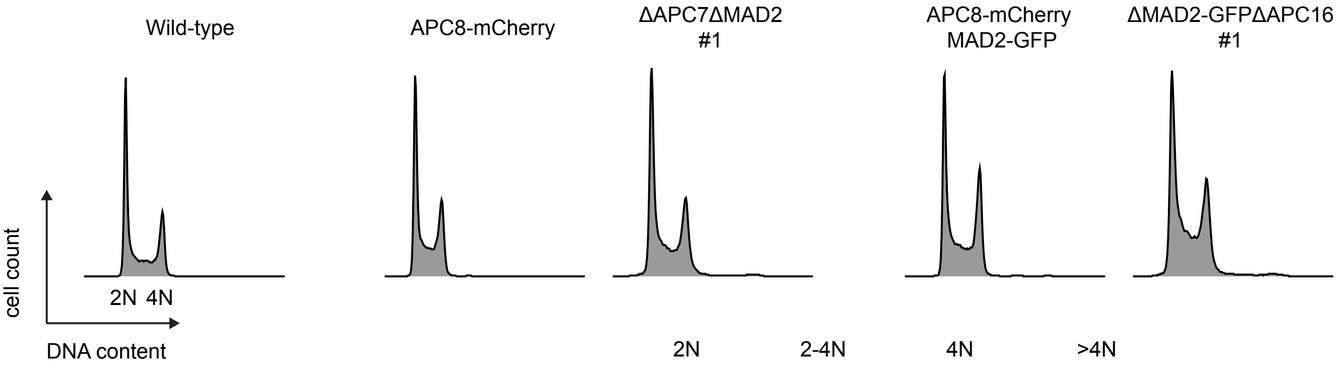

	2N	2-4N	4N	>4N
Wild-type	35 ± 2	43 ± 2	18 ± 1	2 ± 0.3
APC8-mCherry	33 ± 1	44 ± 0.1	19 ± 1	2 ± 0.4
ΔAPC7ΔMAD2 (clones 1-4)	30 ± 2	42 ± 2	18 ± 0.4	7 ± 1
APC8-mCherry MAD2-GFP	27 ± 1	49 ± 1	20 ± 1	3 ± 1
ΔMAD2-GFPΔAPC16 (clones 1-4)	26 ± 2	46 ± 1	18 ± 1	7 ± 1

B

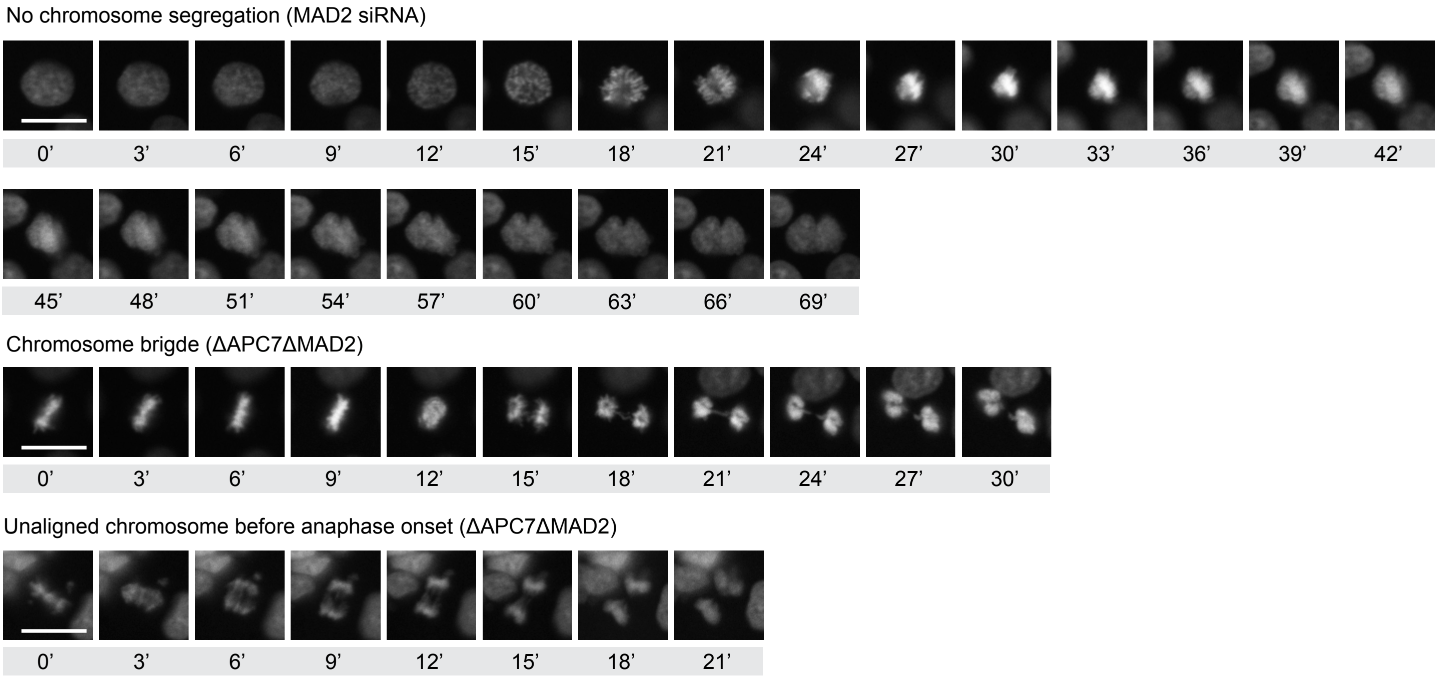

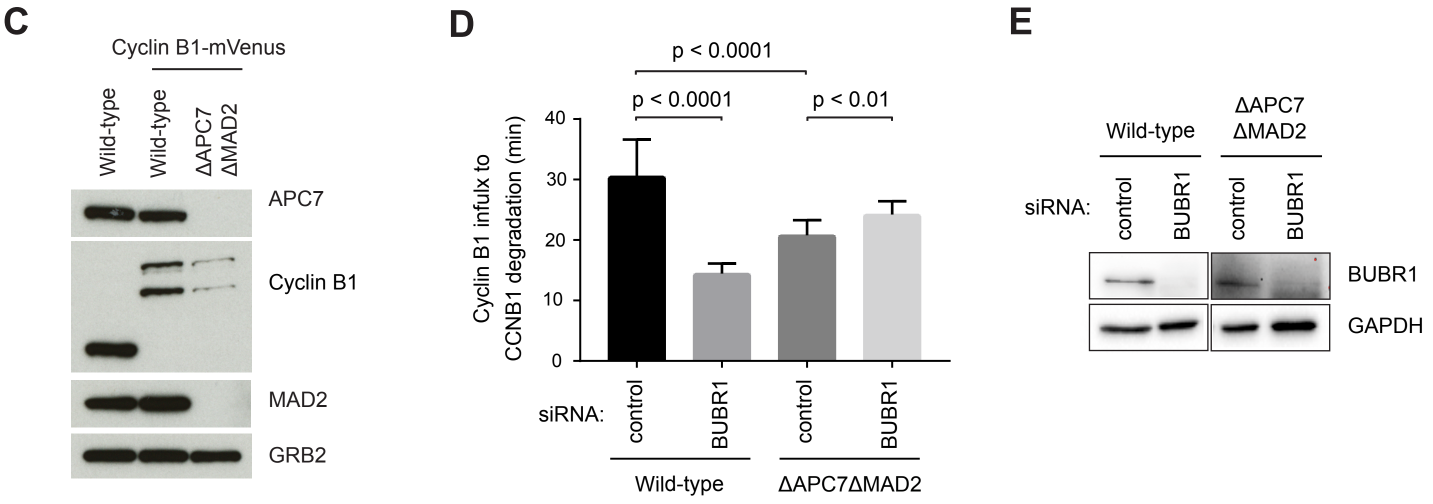

Figure S5. Related to Figure 5.

A) Analysis of DNA content in ΔAPC7ΔMAD2 and ΔMAD2-GFPΔAPC16 clonal cell lines cells. The cellular DNA from wild-type, APC8-mCherry, ΔAPC7ΔMAD2, APC8-mCherry MAD2-GFP, and ΔMAD2-GFPΔAPC16 clonal cell lines was stained with propidium iodide and analysed by FACS. The table shows the percentage of cells with the respective (2N, 2N-4N, 4N and >4N) DNA content.

B) Representative images of chromosome segregation errors summarized in Figure 5C. The scale bars indicate 10 μm. Time points (in minutes) are listed in the grey bar below the images.

C) Immunoblot analysis of the indicated cell lines, used in Figure 5E, S5D, S5E. GRB2 levels were analysed to control for equal loading.

D) Timing form nuclear cyclin B1 import to cyclin B1 degradation at centrosomes from the indicated cell lines and conditions. Live cell imaging was performed 72 hours after siRNA transfection. At least 10 cells per condition were analysed. Error bars depict the standard deviation of the measured timings.

E) Immunoblot analysis of BUBR1 depletion by RNAi in the indicated cell lines, used in D). GAPDH levels were analysed to control for equal loading.
