# Supplementary material for: Deletion of APC7 or APC16 Allows Proliferation of Human Cells without the Spindle Assembly Checkpoint
Source: Cell Rep. 2018 Nov 27;25(9):2317–2328.e5. doi: 10.1016/j.celrep.2018.10.104 (PMC6289045; doi:10.1016/j.celrep.2018.10.104)
Supplement: Document S2. Article plus Supplemental Information [file mmc5.pdf]

Cell Reports

Report

Deletion of APC7 or APC16 Allows Proliferation of Human Cells without the Spindle Assembly Checkpoint

Graphical Abstract

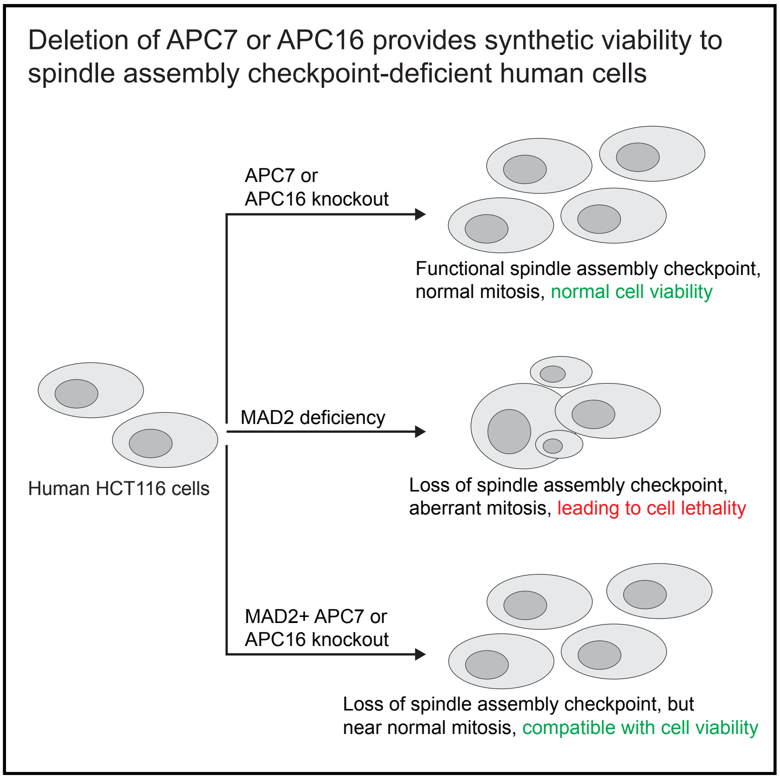

Highlights

- APC16 is required for *in vivo* assembly of APC7 into APC/C
- APC7 or APC16 deletion has no major effect on mitosis
- Deletion of APC7 or APC16 provides synthetic viability to MAD2 deletion

Authors

Thomas Wild, Magda Budzowska, Susanne Hellmuth, ..., Marin Barisic, Olaf Stemmann, Chunaram Choudhary

Correspondence

chuna.choudhary@cpr.ku.dk

In Brief

Anaphase-promoting complex/cyclosome (APC/C) is an essential regulator of mitosis in eukaryotes. Wild et al. show that the APC/C subunits APC7 and APC16 are not required for mitosis in normal cells. However, genetic deletion of these subunits provides synthetic viability to cells lacking the spindle assembly checkpoint.

Wild et al., 2018, Cell Reports *25*, 2317–2328
November 27, 2018 © 2018 The Authors.
https://doi.org/10.1016/j.celrep.2018.10.104

Cell Reports
Report

OPEN ACCESS
CellPress

Deletion of APC7 or APC16 Allows Proliferation of Human Cells without the Spindle Assembly Checkpoint

Thomas Wild,[1,7] Magda Budzowska,[1,2,7] Susanne Hellmuth,[3] Susana Eibes,[4] Gopal Karemore,[5] Marin Barisic,[4,6] Olaf Stemmann,[3] and Chunaram Choudhary[1,8,*]
[1]Proteomics Program, Novo Nordisk Foundation Center for Protein Research, Faculty of Health and Medical Sciences, University of Copenhagen, Blegdamsvej 3B, 2200 Copenhagen, Denmark
[2]Center for Chromosome Stability (CCS), Faculty of Health and Medical Sciences, University of Copenhagen, Blegdamsvej 3B, 2200 Copenhagen, Denmark
[3]Chair of Genetics, University of Bayreuth, Universitätsstraße 30, 95440 Bayreuth, Germany
[4]Danish Cancer Society Research Center, Cell Division Laboratory, Strandboulevarden 49, 2100 Copenhagen, Denmark
[5]Protein Imaging Platform, Novo Nordisk Foundation Center for Protein Research, Faculty of Health and Medical Sciences, University of Copenhagen, Blegdamsvej 3B, 2200 Copenhagen, Denmark
[6]Department of Cellular and Molecular Medicine, Faculty of Health Sciences, University of Copenhagen, Copenhagen, Denmark
[7]These authors contributed equally
[8]Lead Contact
*Correspondence: chuna.choudhary@cpr.ku.dk
https://doi.org/10.1016/j.celrep.2018.10.104

SUMMARY

The multisubunit ubiquitin ligase APC/C (anaphase-promoting complex/cyclosome) is essential for mitosis by promoting timely degradation of cyclin B1. APC/C is tightly regulated by the spindle assembly checkpoint (SAC), which involves MPS1 and MAD2-dependent temporal inhibition of APC/C. We analyzed the contribution of the APC/C subunits APC7 and APC16 to APC/C composition and function in human cells. APC16 is required for APC7 assembly into APC/C, whereas APC16 assembles independently of APC7. APC7 and APC16 knockout cells display no major defects in mitotic progression, cyclin B1 degradation, or SAC response, but APC/C lacking these two subunits shows reduced ubiquitylation activity *in vitro*. Strikingly, deletion of APC7 or APC16 is sufficient to provide synthetic viability to MAD2 deletion. ΔAPC7ΔMAD2 cells display accelerated mitosis and require SAC-independent MPS1 function for genome stability. These findings reveal that the composition of APC/C critically influences the importance of the SAC in humans.

INTRODUCTION

Eukaryotic cells depend on the activity of the anaphase-promoting complex/cyclosome (APC/C), a multi-subunit ubiquitin ligase, to progress through mitosis (Pines, 2011; Primorac and Musacchio, 2013). Among a variety of mitotic APC/C substrates, only securin and cyclin B1 are essential for mitotic progression in yeast (Thornton and Toczyski, 2003). APC/C requires the co-activator CDC20 and an E2 enzyme, UBE2C or UBE2D, to initiate cyclin B1 ubiquitylation (Hershko et al., 1994; Townsley et al., 1997; Wild et al., 2016; Yu et al., 1996). After transferring initial ubiquitin onto substrates, APC/C functions with UBE2S to promote K11-linked polyubiquitylation, which enhances substrate degradation efficiency (Garnett et al., 2009; Meyer and Rape, 2014; Min et al., 2015; Williamson et al., 2009; Wu et al., 2010). Lowering APC/C activity delays mitotic progression, whereas loss of APC/C activity is lethal (Magnuson and Epstein, 1984; Wirth et al., 2004).

The timing of APC/C-initiated securin and cyclin B1 degradation is of critical importance for the faithful segregation of chromosomes. Proper timing is achieved by the spindle assembly checkpoint (SAC), which coordinates bipolar chromosome attachment to the mitotic spindle with APC/C-mediated securin and cyclin B1 ubiquitylation (London and Biggins, 2014; Sivakumar and Gorbsky, 2015). The SAC is activated by recruitment of MPS1 to unattached kinetochores (Hiruma et al., 2015; Ji et al., 2015). Subsequently, MPS1-catalyzed phosphorylation events recruit SAC proteins to unattached kinetochores and promote formation of the mitotic checkpoint complex (MCC), consisting of MAD2, CDC20, BUBR1, and BUB3 (Sudakin et al., 2001). MCC binding to APC/C blocks substrate recognition by CDC20 and hampers access to UBE2C (Alfieri et al., 2016; Yamaguchi et al., 2016). Although SAC function is conserved from yeast to human, its activity is of different importance in different organisms; deletion of SAC proteins, such as MAD2, is compatible with viability in yeast and *Drosophila* but leads to severe genomic instability in mice and human cells that is incompatible with life (Buffin et al., 2007; Dobles et al., 2000; Kops et al., 2005; Meraldi et al., 2004; Michel et al., 2001; 2004).

The human APC/C consists of 19 subunits composed of 14 distinct proteins. The atomic architecture of APC/C reveals that APC1 and APC2 form the core of the platform, whereas the tetratricopeptide repeat (TPR) subunits APC3, APC6,

This is an open access article under the CC BY license (http://creativecommons.org/licenses/by/4.0/).

A

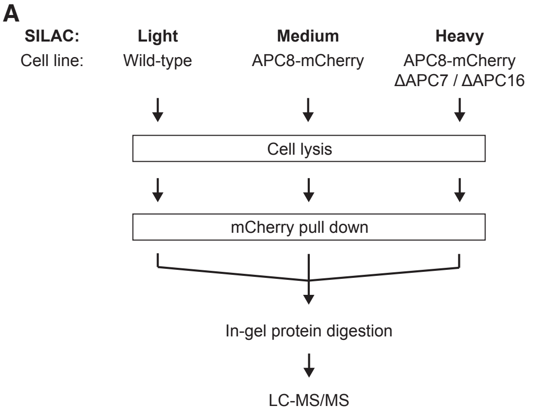

B

APC subunits	SILAC ratio (APC / Ctr)	($APC^{\Delta APC7}$/ Ctr)	($APC^{\Delta APC7}$ / APC)
APC1	25.18	26.20	1.06
APC2	25.60	25.29	1.00
APC3	17.53	20.03	1.12
APC4	19.98	21.46	1.06
APC5	19.34	21.23	1.07
APC6	19.58	21.37	1.08
APC7	40.15	n.d.	n.d.
APC8	16.65	20.91	1.25
APC10	22.13	24.07	1.07
APC12	31.06	32.59	1.08
APC13	23.05	25.71	1.15
APC15	23.40	24.69	1.07
APC16	19.04	16.50	0.84

C

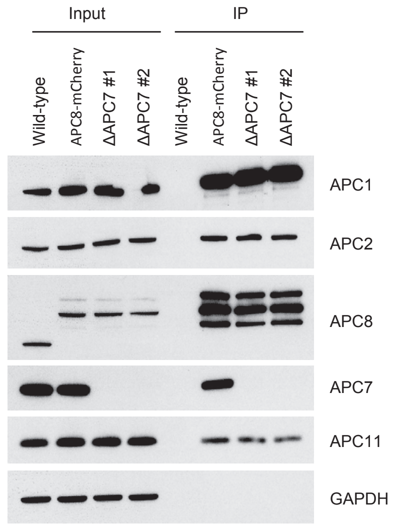

D

APC subunits	SILAC ratio (APC / Ctr)	($APC^{\Delta APC16}$/ Ctr)	($APC^{\Delta APC16}$ / APC)
APC1	20.71	26.39	1.27
APC2	22.39	22.88	1.01
APC3	21.58	18.00	0.83
APC4	20.15	24.26	1.17
APC5	19.42	22.88	1.14
APC6	21.84	20.45	0.90
APC7	14.21	0.14	0.01
APC8	14.64	24.24	1.63
APC10	23.30	20.99	0.79
APC12	26.64	21.55	0.84
APC13	14.60	21.04	1.40
APC15	16.05	29.51	1.91
APC16	34.43	n.d.	n.d.

E

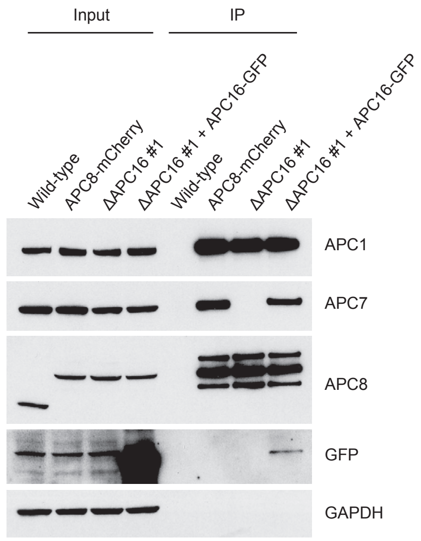

(legend on next page)

APC7, and APC8 constitute the majority of the "arc lamp" (Chang et al., 2015). The catalytic center of APC/C is formed by APC11 and APC2 along with APC10 and the co-activators CDC20 or CDH1 for substrate recognition. APC/C composition is conserved from yeast to human, except for the two subunits, APC7 and APC16, located at the tip of the arc lamp (Chang et al., 2015). APC7 is present in two copies and, together with one APC16 molecule, sits on top of APC3. APC16 is implicated in mitotic progression and APC/C substrate stability but not APC/C assembly (Kops et al., 2010; Shakes et al., 2011). Depletion of APC7 in *Drosophila melanogaster* had a limited effect on mitotic progression, and an APC7 null strain is viable (Pál et al., 2007). *In vitro*, it has been shown that APC3 forms a sub-complex with APC7 and that this sub-complex is stabilized by APC16 (Yamaguchi et al., 2015). Based on these data, the authors suggest that the APC3/APC7/APC16 sub-complex may constitute an assembly unit or that APC16 recruits APC7 onto APC3. Here we set out to analyze the contribution of APC7 and APC16 to APC/C assembly, function, and regulation using human genetic knockouts cells.

RESULTS

APC16 Is Required for APC7 Assembly into APC/C *In Vivo*

To study the effect of APC7 and APC16 on the composition of APC/C *in vivo*, we fused mCherry to the C terminus of APC8 and investigated APC/C composition by affinity purification mass spectrometry (AP-MS). Because APC8 is essential for cell viability, biallelic tagging of the gene indicated functional integrity of APC8-mCherry-tagged APC/C. Next, we individually deleted APC7 and APC16 in the APC8-mCherry cell line (Figures S1A and S1B) and analyzed APC/C composition with stable isotope labeling by amino acids in cell culture (SILAC)-based AP-MS (Figure 1A). We purified APC/C from wild-type and ΔAPC7 cells by APC8-mCherry affinity purification. In wild-type cells, all detected APC/C subunits were enriched (>2-fold) (Figure 1B; Table S1), demonstrating proper incorporation of APC8-mCherry into APC/C complexes. All APC/C subunits, except for APC7, co-purify with APC8-mCherry from ΔAPC7 cells, showing that the remaining APC/C subunits assemble in the absence of APC7 (Figure 1B). We confirmed APC/C assembly in ΔAPC7 cells by immunoblotting (Figure 1C). We used a similar AP-MS approach to analyze the role of APC16 in APC/C assembly. Interestingly, APC/C enriched from ΔAPC16 cells not only lacked APC16 but also APC7 (Figure 1D; Figure S1C; Table S1). To test whether lack of APC7 was due to the loss of APC16, we expressed APC16-EGFP in ΔAPC16 cells and analyzed APC/C composition (Figure 1E). Consistent with its integration into APC/C (Kops et al., 2010), APC16-EGFP co-purified with APC8-mCherry and, indeed, restored APC7 co-purification with APC8-mCherry. Together, these results show that, *in vivo*, APC16 incorporates into APC/C in the absence of APC7, whereas APC7 requires APC16 for incorporation into APC/C.

APC/C Lacking APC7 and APC16 Are Less Active *In Vitro*

We next tested whether APC/C lacking APC7 or APC7 and APC16 display altered ubiquitylation activity. For this, we immunoprecipitated APC/C from wild-type, ΔAPC7, and ΔAPC16 cells in an anaphase-like state and analyzed APC/C activity, in the presence of the two E2 enzymes UBE2C and UBE2S, toward securin (Hellmuth et al., 2014). Compared with wild-type APC/C, a decrease in higher molecular securin species was observed upon incubation with APC/C purified from either ΔAPC7 or ΔAPC16 cells (Figures 2A and 2B; Figure S1D). Similarly, APC/C purified from ΔAPC7 and ΔAPC16 cells showed reduced ubiquitylation activity toward cyclin B1, particularly apparent at longer incubation times (Figures 2C and 2D; Figure S1E). Overall, these results revealed slightly reduced ubiquitylation activity of APC/C lacking APC7 or APC7 and APC16 toward two of its substrates, securin and cyclin B1, *in vitro*.

Cells Lacking Either APC7 or APC16 Display No Major Defects in Mitotic APC/C Function

To analyze the role of APC7 and APC16 in mitotic progression, we endogenously tagged histone H2B with mVenus and cyclin B1 with mCerulean3 in the wild-type, ΔAPC7, and ΔAPC16 background (Figures S2A–S2C) and analyzed mitotic timing by time-lapse microscopy. No significant difference was observed for mitotic timing (defined as the timing from nuclear cyclin B1 influx to anaphase onset) between wild-type, ΔAPC7, and ΔAPC16 cells (Figure 3A; Figure S2C). Concordantly, no significant alteration was found in the kinetics of mitotic cyclin B1 degradation between wild-type and APC7 or APC16 knockout cells (Figure 3B). Hence, the slightly reduced APC/C activity

Figure 1. APC16 Is Required for Assembly of APC7 into APC/C

(A) Experimental setup for MS-based analysis of APC/C composition. APC8-mCherry-expressing wild-type cells were labeled with medium SILAC, and ΔAPC7 or ΔAPC16 cells were labeled with heavy SILAC. APC/C was purified using mCherry affinity beads, and as a control, a mock pull-down was performed from light SILAC-labeled cells.

(B) SILAC ratios for APC/C subunits enriched from APC8-mCherry wild-type and APC8-mCherry ΔAPC7 cells. APC/C was purified with mCherry pull-downs from wild-type (medium SILAC) and ΔAPC7 cells (heavy SILAC) and analyzed by MS. Mock pull-down from wild-type HCT116 cells served as a control (light SILAC). The table shows combined SILAC ratios for APC/C subunits detected in three technical replicates (n.d., not determined).

(C) Analysis of APC/C composition in ΔAPC7 cells. APC/C was purified via APC8-mCherry pull-downs from APC8-mCherry and APC8-mCherry ΔAPC7 cells (from two independent ΔAPC7 clonal cell lines) and subsequently analyzed by immunoblotting using the indicated antibodies. Wild-type cells were used as a control for unspecific binding to the affinity beads. GAPDH levels were analyzed to verify equal amounts of input for the different cell lines.

(D) SILAC ratios for APC/C subunits enriched from APC8-mCherry and APC8-mCherry ΔAPC16 cells. The analysis was performed as described in (B).

(E) Immunoblot analysis of APC/C composition in ΔAPC16 cells with the indicated antibodies. APC/C was purified via APC8-mCherry pull-downs from APC8-mCherry cells and from APC8-mCherry ΔAPC16 cells. The indicated samples were transiently transfected with APC16-EGFP 32 hr prior to APC/C pull-down. Wild-type cells were used as a control for unspecific binding to the affinity beads.

See also Figure S1.

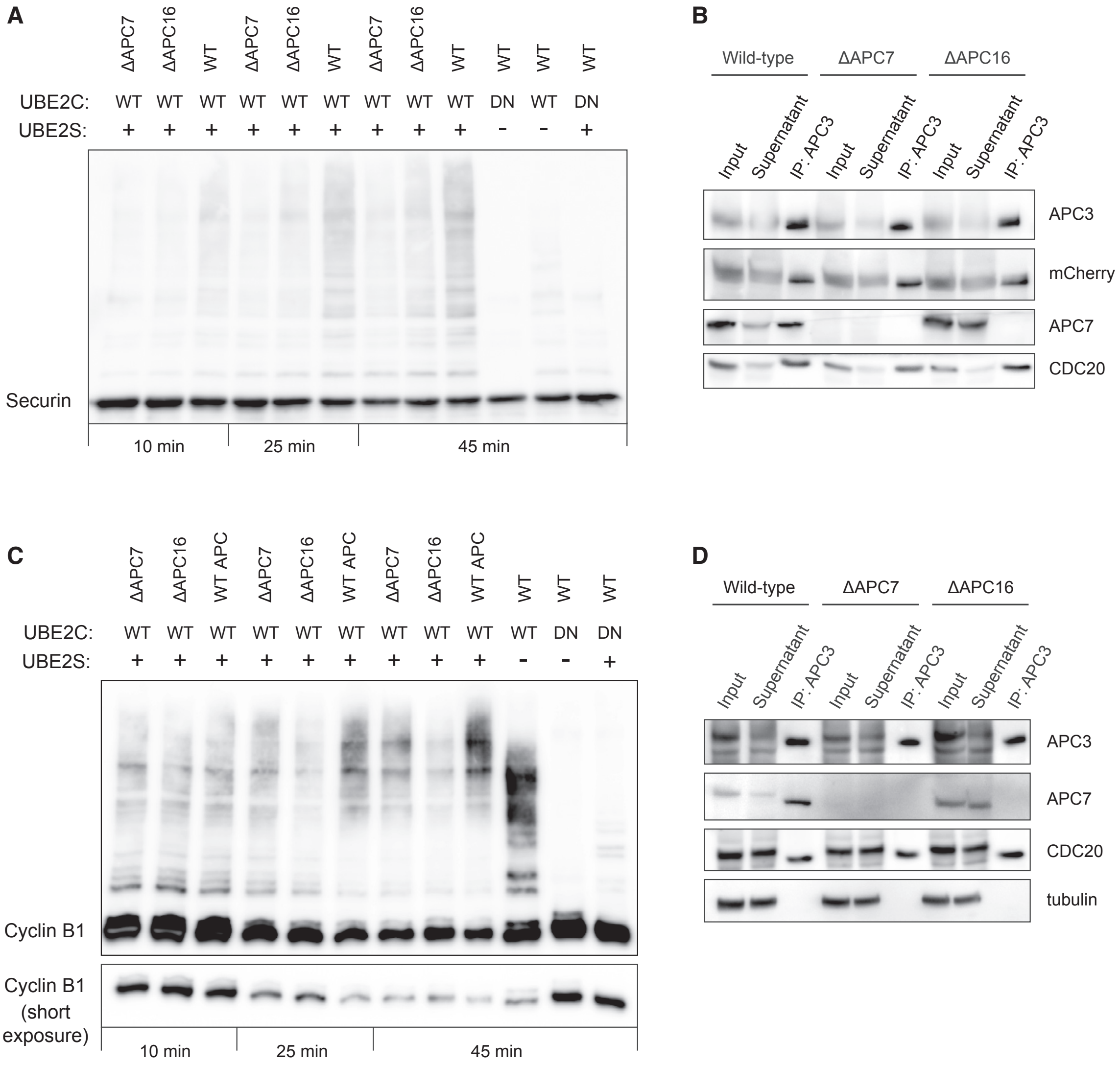

Figure 2. Analysis of *In Vitro* Ubiquitylation Activities of APC/C Lacking APC16 and/or APC7

(A) Ubiquitylation activity of purified APC/C variants toward securin. APC/C was purified from APC8-mCherry (WT), ΔAPC7 APC8-mCherry (ΔAPC7), or ΔAPC16 APC8-mCherry (ΔAPC16) cells using an antibody against APC3 and incubated for different times with recombinant securin supplemented with UBE2C and UBE2S as indicated. Ubiquitylation of securin was analyzed by an α-securin immunoblot. A representative result from two experiments is shown. WT, wild-type; DN, dominant-negative.

(B) Immunoblot analysis of the purified APC/C used for Figure 2A. Input, supernatant, and immunoprecipitated fractions (immunoprecipitation [IP]: APC3) from the indicated cell lines were analyzed with the indicated antibodies.

(C) Ubiquitylation activity of purified APC/C variants toward cyclin B1 as described in (A). Ubiquitylation of cyclin B1 was analyzed by an α-cyclin B1 immunoblot. A representative result from two experiments is shown.

(D) Immunoblot analysis of the purification of APC/C used for Figure 2C, analyzed as described in (B).

See also Figure S1.

upon loss of APC7 or APC7 and APC16, measured *in vitro*, does not elicit a discernable effect on mitotic timing and cyclin B1 degradation *in vivo*.

Next, we assessed the responsiveness of ΔAPC7 and ΔAPC16 cells to SAC activation. Cells were treated with nocodazole, and the cellular DNA content was analyzed by flow cytometry (fluorescence-activated cell sorting [FACS]) (Figure S3A). For all analyzed cell lines, nocodazole treatment caused a marked increase in the number of cells with 4N DNA, showing that ΔAPC7 and ΔAPC16 cells remain sensitive to SAC signaling. To further verify SAC functionality in ΔAPC7 cells, we analyzed the nocodazole-dependent association of SAC-generated MCC with APC/C. For both wild-type and ΔAPC7 cells, increased enrichment of the SAC proteins BUB3, BUBR1, CDC20, and MAD2 was observed in APC8-mCherry pull-downs upon nocodazole-induced mitotic arrest (Figures 3C and 3D).

A

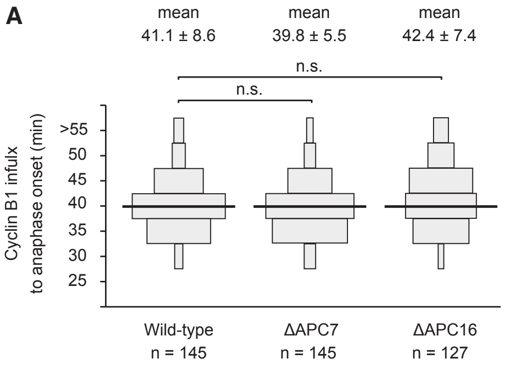

B

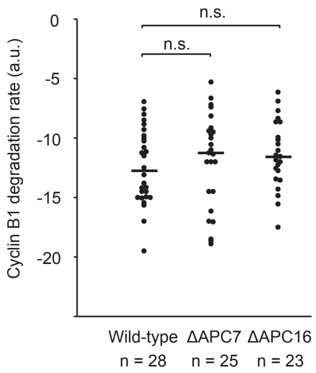

	Mean cyclin B1 degradation rate
Wild type	-12.4 ± 3.1
ΔAPC7	-11.9 ± 3.9
ΔAPC16	-11.4 ± 2.8

C

APC/C

		untreated / ctr	noc / ctr	noc / untreated
APC subunits	APC1	39.99	26.44	0.68
	APC2	38.03	32.36	0.89
	APC3	25.41	22.43	0.91
	APC4	28.16	19.73	0.71
	APC5	26.52	19.30	0.71
	APC6	22.24	20.96	0.92
	APC7	22.78	22.92	1.00
	APC8	26.66	19.31	0.70
	APC10	22.53	22.21	0.93
	APC12	35.95	35.32	0.99
	APC13	40.62	28.47	0.72
	APC15	38.91	27.23	0.69
	APC16	23.19	22.48	0.97
MCC subunits	BUB3	7.68	49.50	6.52
	BUBR1	5.91	38.42	6.63
	CDC20	9.95	58.96	5.79
	MAD2	4.31	30.06	6.26

D

APC/C$^{\Delta APC7}$

		untreated / ctr	noc / ctr	noc / untreated
APC subunits	APC1	30.53	23.25	0.78
	APC2	29.64	26.09	0.89
	APC3	22.72	19.90	0.89
	APC4	24.34	19.40	0.81
	APC5	22.83	19.08	0.82
	APC6	20.95	17.74	0.85
	APC8	24.02	22.29	0.96
	APC10	30.79	29.21	0.90
	APC12	28.92	25.02	0.89
	APC13	25.24	24.38	0.96
	APC15	27.83	22.10	0.79
	APC16	23.28	19.67	0.87
MCC subunits	BUB3	3.02	42.49	14.26
	BUBR1	2.71	42.19	15.34
	CDC20	4.29	48.85	12.07
	MAD2	3.14	41.07	13.56

E

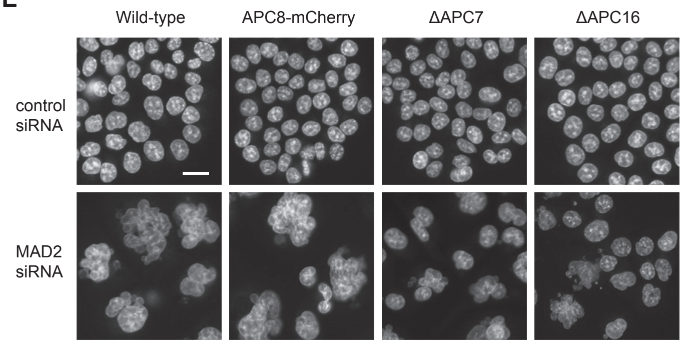

F

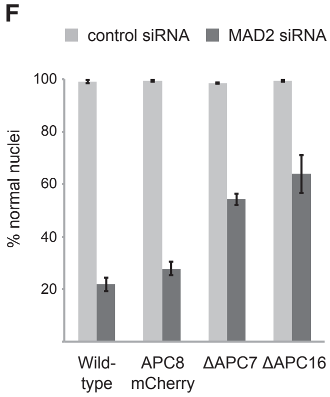

(legend on next page)

We next assayed the robustness of the SAC response in ΔAPC7 and ΔAPC16 cells using live-cell imaging (Figure S3B). These data revealed no difference in SAC robustness between wild-type and ΔAPC16 cells and indicated a slightly less robust SAC in ΔAPC7 cells. To analyze the dependence of ΔAPC7 and ΔAPC16 cells on SAC function for genomic stability, we treated the cell lines with the MPS1 inhibitor reversine and analyzed cellular ploidy by FACS (Figure S3C). Reversine treatment increased the number of polyploid cells (>4N DNA content), demonstrating the importance of MPS1 activity for maintaining genome stability in these cell lines. To independently assess the importance of SAC function in ΔAPC7 and ΔAPC16 cells, we depleted MAD2 by RNAi and analyzed nuclear morphology 4 days thereafter (Figure 3E; Figure S3D). Consistent with the crucial role of MAD2 in maintaining genomic stability, MAD2-depleted wild-type cells showed a marked decrease in normally sized and shaped nuclei (Figure 3F). Interestingly, however, the appearance of enlarged multi-lobed nuclei upon MAD2 RNAi was mitigated in both ΔAPC7 and ΔAPC16 cells (Figure 3F). Overall, these results indicate that loss of APC7 or APC16 has little effect on mitotic progression, APC/C activity toward cyclin B1, and SAC functionality. However, cell lines lacking either APC7 or APC16 appear to rely less on MAD2 for maintaining their genome stability.

Genetic Ablation of MAD2 in APC7 or APC16 Knockout Cells

Because the ΔAPC7 and ΔAPC16 cell lines displayed less severe effect of MAD2 depletion on their genome stability (Figure 3F), we tested whether genetic ablation of MAD2 is tolerated in these cell lines. To assess the synthetic viability of MAD2 deletion with the loss of APC7, we mixed equal numbers (i.e., 1:1:1) of wild-type, APC8-mCherry, and APC8-mCherry ΔAPC7 cells (Figure 4A; Figure S4A) and performed CRISPR-mediated MAD2 deletion in the resulting mixed cell population. After selection, single colonies were analyzed for the absence of MAD2 by immunoblotting. We recovered a total of six ΔMAD2 cell lines, all of which were derived from the APC8-mCherry ΔAPC7 background (Figure 4B). Not retrieving MAD2 knockout clones from wild-type backgrounds is consistent with MAD2 being essential in human cells. Identification of multiple MAD2 knockout cell lines in the ΔAPC7 background demonstrates that cells lacking APC7 can tolerate loss of MAD2. To confirm the observed synthetic viability and to assess whether it also applies to APC16 deletion, we devised an approach facilitating direct deletion of MAD2 from wild-type cells along with co-deletion of either APC7 or APC16. For this, we GFP-tagged endogenous MAD2 and transfected these cells with a MAD2 deletion construct bearing a selection marker as well as with a selection-less plasmid encoding a guide RNA targeting either APC7 or APC16 (Figure 4C; Figure S4B). After selection, cell colonies were isolated based on the visually observed loss of GFP fluorescence (indicating loss of MAD2-GFP), and the absence of MAD2-GFP was subsequently confirmed by immunoblotting. Concurrent loss of APC7 and APC16 was assessed by immunoblotting and genotyping, respectively. Using this approach, we obtained four ΔMAD2-GFPΔAPC7 and six ΔMAD2-GFPΔAPC16 cell lines (Figure 4D; Figures S4C and S4D). Because the experimental setup only selects for MAD2 deletion, the strict co-deletion of either APC7 or APC16 with MAD2 deletion indicates that the observed viability is synthetic. Consistent with an essential role of MAD2 in SAC activity, neither ΔAPC7ΔMAD2 nor ΔMAD2-GFPΔAPC16 cells arrested in mitosis upon nocodazole treatment (Figure 4E; Figure S4E). Together, these results show that deletion of either APC7 or APC16 allows for continued proliferation in the absence of MAD2.

Analysis of Cells Lacking MAD2

Loss of SAC activity leads to genomic instability (Dobles et al., 2000; Kops et al., 2005; Figures 3E and 3F). We therefore analyzed the cellular DNA content of different ΔAPC7ΔMAD2 and ΔMAD2-GFPΔAPC16 clonal cell lines by FACS (Figure S5A). A modest increase in polyploid cells was observed in the SAC-deficient cell lines compared with wild-type cells, indicating ongoing but tolerable chromosomal instability. To further

Figure 3. Analysis of Mitotic APC/C Function in ΔAPC7 and ΔAPC16 Cells

(A) Time between nuclear cyclin B1 influx and anaphase onset for the indicated cell lines. Wild-type, ΔAPC7, and ΔAPC16 cell lines expressing mVenus-tagged histone H2B and mCerulean3-tagged cyclin B1 were imaged every 5 min. The data show the time from nuclear cyclin B1 influx to anaphase onset for individual cells. Cells with identical timing are depicted as a box, and the size of each box is scaled according to the percentage they contribute to the whole population of the respective cell line. The mean timing and SD are stated on the top. The median timing is drawn as a black line. Results of three independent experiments are shown, and the number (n) of analyzed cells is stated for each cell line. A two-tailed t test was performed to calculate significance ($p < 0.01$ = significant; n.s., non-significant).

(B) Cyclin B1 degradation rates around anaphase onset in the indicated cell lines (data from the experiment shown in A). The plot shows cyclin B1 degradation rates for individual cells from the indicated genetic backgrounds. Cyclin B1 degradation rates in individual cells were calculated by the slope of a linear fit for the measured decrease in cyclin B1-mCerulean3 intensities around anaphase onset. The number of analyzed cells is given as n. The median cyclin B1 degradation rate is drawn as a black line. A two-tailed t test was performed to calculate significance ($p < 0.01$ = significant). The table on the right shows the mean cyclin B1 degradation rate and its SD in the indicated cell lines.

(C) SILAC ratios for APC/C and MCC subunits detected in MS analysis of APC8-mCherry pull-down with or without SAC activation. APC/C was purified via APC8-mCherry pull-downs from untreated cells (medium SILAC) and from cells treated with 200 nM nocodazole (noc) for 18 hr (heavy SILAC). Depicted are the combined SILAC ratios from three technical replicates. A control pull-down from light SILAC was used as a reference for unspecific binding to the affinity beads.

(D) SILAC ratios for APC/C and MCC subunits detected in MS analysis of APC8-mCherry pull-down from APC8-mCherry ΔAPC7 cells, analyzed as described in (C).

(E) The indicated cell lines were treated with control small interfering RNA (siRNA) or MAD2-targeting siRNA for 96 hr, stained with Hoechst, and imaged. Representative images from one of the three independent experiments are shown. The scale bar indicates 10 μm.

(F) Quantification of normal nuclei from the experiments described in (E). From each experiment, at least 500 cells were analyzed per condition, and the cumulative percentage of normal nuclei from all three experiments is shown. Error bars depict SD from 3 experiments.

See also Figures S2 and S3.

OPEN ACCESS
CellPress

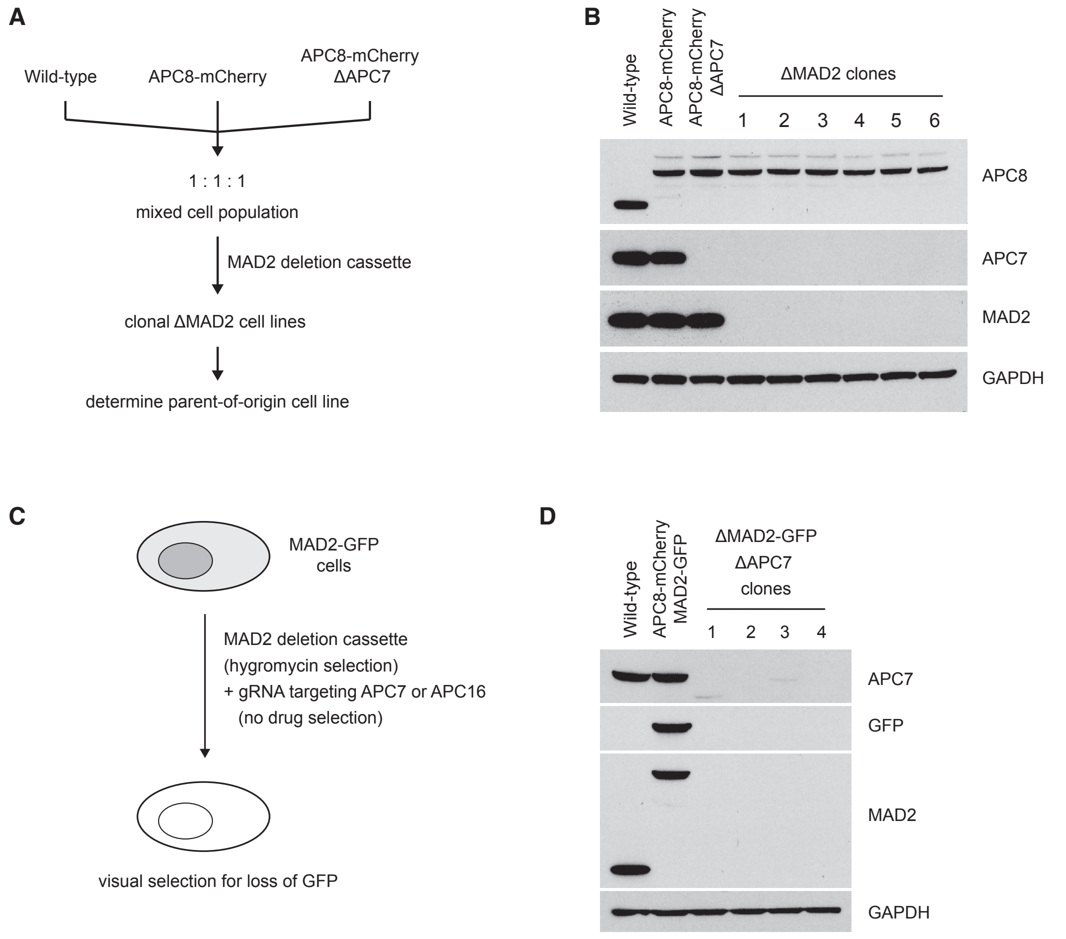

E

Wild-type + control RNAi

	noc (-)	noc (+)
2N	53	2
2-4 N	20	3
4N	25	92
>4N	2	3

Wild-type + MAD2 RNAi

	noc (-)	noc (+)
2N	25	10
2-4 N	18	11
4N	33	47
>4N	23	31

ΔAPC7ΔMAD2

	noc (-)	noc (+)
2N	42	9
2-4 N	23	6
4N	29	48
>4N	5	38

ΔMAD2-GFPΔAPC16

	noc (-)	noc (+)
2N	37	6
2-4 N	25	6
4N	31	45
>4N	6	43

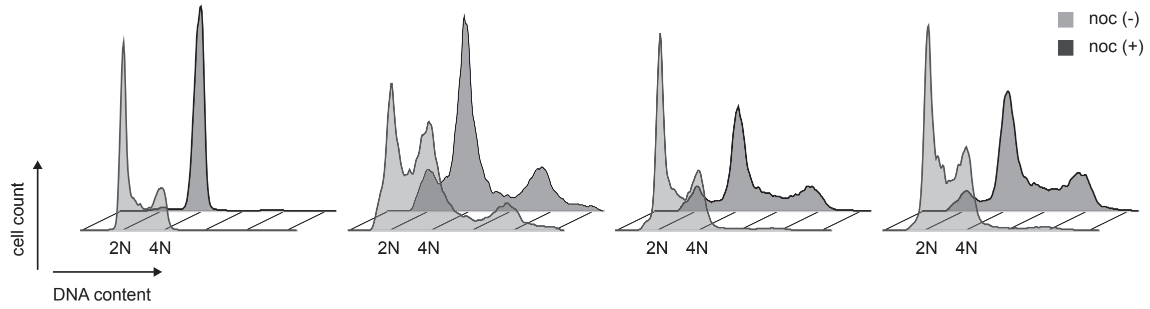

(legend on next page)

explore the genomic stability of ΔAPC7ΔMAD2 cells, we compared mitosis and chromosome segregation in wild-type, ΔAPC7ΔMAD2, and wild-type cells depleted of MAD2 by RNAi. To this end, we fluorescently tagged histone H2B in wild-type and ΔAPC7ΔMAD2 cells and analyzed them by live-cell imaging (Figure 5A). We observed a decreased time from chromatin condensation to anaphase onset in ΔAPC7ΔMAD2 cells compared with wild-type cells, consistent with the function of MAD2 in delaying mitotic progression in wild-type cells (Figure 5B). Wild-type cells depleted of MAD2 by RNAi most frequently (85%) proceeded through mitosis without formation of a metaphase plate, resulting in aberrant or undetectable chromosome segregation (Figures 5A and 5C; Figure S5B). In contrast, ΔAPC7ΔMAD2 cells frequently (96%) formed a metaphase plate despite lacking MAD2 function and segregated chromosomes (Figures 5A and 5C). Compared with wild-type cells, we captured more chromosome segregation errors in ΔAPC7ΔMAD2 cells (Figure 5C; Figure S5B), indicating an increased rate of ongoing segregation errors.

Because MPS1 acts upstream of MAD2 in the SAC response, we tested whether ablation of MAD2 would relieve cells of the need for MPS1 kinase activity to maintain their genome stability. FACS analysis of reversine-treated ΔAPC7ΔMAD2 or ΔMAD2-GFPΔAPC16 cells showed a marked increase in cells with more than 4N DNA content (Figure 5D). To analyze the effect of reversine on cyclin B1 degradation in ΔAPC7ΔMAD2 cells, we fluorescently tagged cyclin B1 in this background along with a corresponding wild-type control (Figure S5C). Consistent with the mitotic timing shown in Figure 5B, cyclin B1 degradation was accelerated in ΔAPC7ΔMAD2 cells compared with wild-type cells (Figure 5E). As expected, reversine treatment significantly accelerated the onset of cyclin B1 degradation in wild-type cells. In contrast, reversine did not accelerate cyclin B1 degradation in ΔAPC7ΔMAD2 cells. Hence, MAD2 deletion eliminates the mitotic timing function of MPS1 but does not relieve these cell lines of the need for MPS1 activity to prevent excessive polyploidy. Consistent with the SAC independence of ΔAPC7ΔMAD2 cells, BUBR1 depletion by RNAi did not accelerate cyclin B1 degradation in these cells, in marked contrast to wild-type cells (Figures S5D and S5E). In summary, cells lacking MAD2 and APC7 display accelerated mitosis concurrent with increased chromosome segregation errors and require MPS1 activity for genome stability in the absence of a functional SAC.

DISCUSSION

APC/C is the largest known ubiquitin ligase in human cells. How this complex assembles *in vivo* is not fully understood. Our work shows that APC16 is required for APC7 assembly into APC/C *in vivo* and that APC16 can incorporate into APC/C independent of APC7. Gratifyingly, these results are in line with *in vitro* data on an APC3/APC7/APC16 sub-complex (Yamaguchi et al., 2015).

Analysis of key aspects of APC/C function, namely mitotic timing, cyclin B1 degradation, and response to spindle assembly defects, revealed no significant alterations upon loss of either APC7 or APC16. It has been reported previously that RNAi-based depletion of APC16 in HeLa cells and *C. elegans* results in mitotic defects (Kops et al., 2010). It is currently unclear why APC16 knockdown and APC16 deletion result in different phenotypes, but the difference between acute and permanent loss may contribute to this discrepancy. Another possibility is that cells require stronger activity of APC/C in polyploid cells compared with haploid or diploid cells cultured *in vitro*.

The major phenotype detected in APC7 or APC16 knockout cells is their reduced dependence on MAD2 function for viability, indirectly revealing a functional effect of these APC/C subunits in HCT116 cells. The penetrance of genome instability upon MAD2 depletion by RNAi is significantly reduced in ΔAPC7 and ΔAPC16 cells compared with wild-type HCT116 cells. We speculate that this reduced dependence on MAD2 function ultimately allows tolerance of MAD2 deficiency in the ΔAPC7 and ΔAPC16 cell lines. The synthetic viability between the loss of MAD2 and deletion of either APC7 or APC16 may be particularly notable given that no measurable increase is observed in the mitotic length in ΔAPC7 and ΔAPC16 cells. Our previous work showed that deletion of two APC/C-employed E2 enzymes, UBE2S and UBE2C, provides viability to MAD2 deletion (Wild et al., 2016). ΔUBE2SΔUBE2C cells display significantly prolonged mitosis, and it was therefore rationalized that it is this increased length of mitosis that enables cells to properly segregate chromosomes

Figure 4. Loss of APC7 or APC16 Provides Synthetic Viability to MAD2 Deletion

(A) Strategy for assessing the synthetic viability of MAD2 deletion with the ΔAPC7 genotype. Equal numbers of wild-type, APC8-mCherry, and APC8-mCherry ΔAPC7 cells were mixed and seeded together, resulting in a mixed cell population with one-third of cells lacking APC7. The genomic MAD2 locus was then targeted by CRISPR/Cas9 using a drug-selectable donor plasmid designed to disrupt the MAD2 gene. After selection, each clonal cell line was analyzed by immunoblotting to assess loss of MAD2 and to determine its parent-of-origin cell line.

(B) Analysis of APC7 and MAD2 expression in the six clonal ΔMAD2 cell lines retrieved from the setup outlined in (A). GAPDH levels served as a loading control. Note that all six retrieved clonal cell lines derive from APC8-mCherry ΔAPC7 cells.

(C) Scheme of the CRISPR/Cas9-based MAD2-GFP synthetic viability assay. Cells expressing endogenously GFP-tagged MAD2 (MAD2-GFP) were transfected with a drug-selectable deletion cassette for the MAD2 gene along with a drug selection-less plasmid encoding a guide RNA targeting either APC7 or APC16. After drug selection, cell colonies were microscopically inspected for loss of MAD2-GFP fluorescence. Cell colonies lacking green fluorescence were then analyzed by immunoblotting for loss of MAD2 expression. The status of APC7 and APC16 in MAD2-deficient cells was assessed by immunoblotting or sequencing of the genomic locus, respectively.

(D) Immunoblot analysis of the four clones retrieved from the MAD2-GFP synthetic viability assay performed in combination with an APC7 targeting guide RNA. Note that all retrieved clonal cell lines lost expression of APC7.

(E) Analysis of SAC functionality in ΔAPC7ΔMAD2 and ΔMAD2-GFPΔAPC16 cells. Cellular DNA from wild-type, ΔAPC7ΔMAD2, and ΔMAD2-GFPΔAPC16 cells, with or without 18 hr of 200 nM nocodazole, was stained with propidium iodide and analyzed by flow cytometry. MAD2 depletion by RNAi was performed in wild-type HCT116 cells for comparison. The tables at the top show the percentage of cells with the respective (2N, 2N-4N, 4N, and >4N) DNA content. The bottom panel shows the corresponding flow cytometry profiles from the indicated cell lines.

See also Figure S4.

A

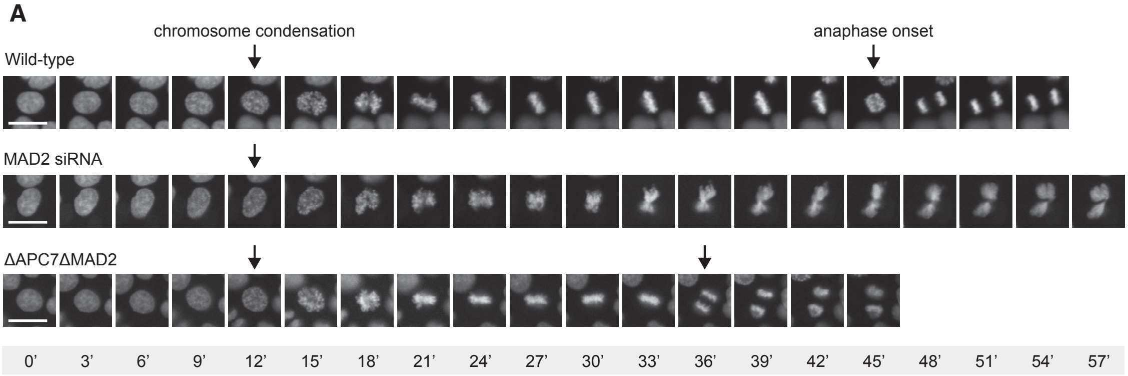

B

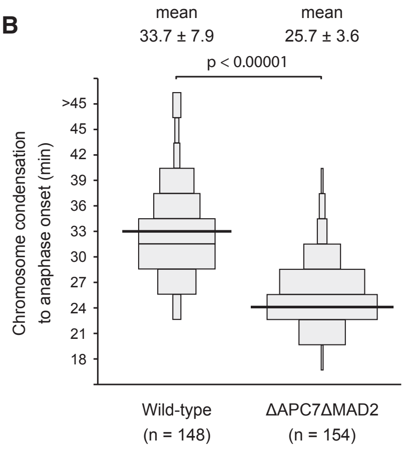

C

	Wild type	ΔAPC7 ΔMAD2	Wild type + MAD2 RNAi
Normal mitosis (number of cells / % cells)	148 / 100%	140 / 89%	18 / 13%
Aberrant mitosis without metaphase plate:			
without chromosome separation	0	4	39
with chromosome separation	0	2	76
Aberrant mitosis with metaphase plate formation:			
chromosome bridge after anaphase onset	0	10	2
unaligned chromosome before anaphase onset	0	1	0
Total errors (number of cells / % cells)	0 / 0%	17 / 11%	117 / 87%

D

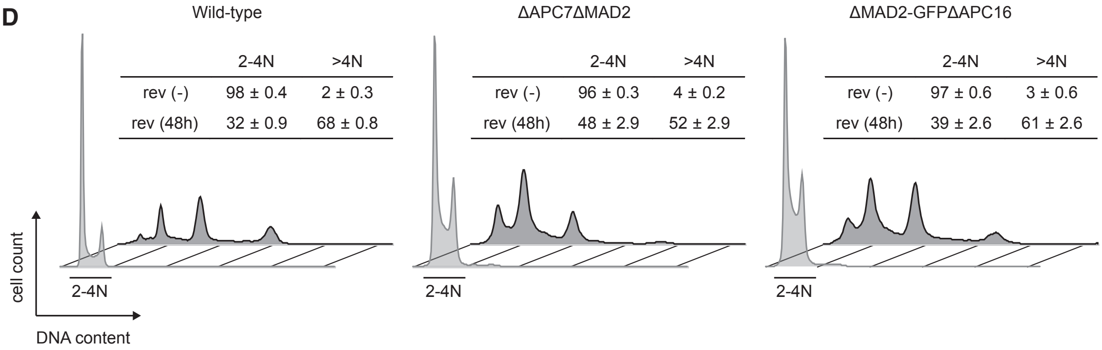

E

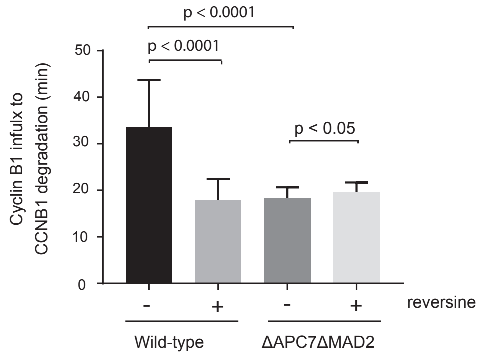

(legend on next page)

in the absence of SAC activity. In contrast, mitosis was not detectably prolonged in ΔAPC7 and ΔAPC16 cells, but these genetic backgrounds provide synthetic viability to MAD2 deletion. Therefore, even a mild reduction in APC/C activity, which may be below the detection limit of our live-cell assay, may suffice to render MAD2 function non-essential in ΔAPC7 and ΔAPC16 cells. This idea is supported by our analysis of APC/C activity *in vitro*, which revealed a small but discernible decrease in APC/C activity when APC7 or APC7 and APC16 are missing. It is possible that such small alterations in APC/C activity do not critically affect unperturbed mitosis in cultured cells but that they become decisive when the SAC is weakened or lost. Alternatively, other, yet to be discovered mechanisms to sustain genome stability in the absence of SAC signaling may exist. Interestingly, a recent study revealed that several SAC genes are not essential in the near-haploid human cell line HAP1 even though APC/C activity appears to be unaltered in these cells (Raaijmakers et al., 2018). Other instances of viability in the absence of MAD2 have been reported in murine and human cells (Burds et al., 2005; Foijer et al., 2017; 2013), and it will be of interest to dissect the properties of these MAD2 knockouts.

Both MPS1 and MAD2 act to maintain the stable euploid state of eukaryotic genomes. In SAC signaling, MPS1 functions upstream of MAD2, but it also has established functions outside of SAC signaling (Liu and Winey, 2012). We find that cells lacking MAD2 and, thus, SAC function still require MPS1 for maintaining their genome stability. Hence, our results indicate that SAC-independent MPS1 functions, such as its role in chromosome alignment, are essential for maintaining genome integrity. Because aneuploidy is frequently detected in human cancers (Funk et al., 2016; Kops et al., 2005), understanding the mechanisms controlling genome stability, such as the SAC, will advance our knowledge of mechanisms preventing genome instability and mechanisms underlying an increased tolerance toward ongoing genome instability.

STAR★METHODS

Detailed methods are provided in the online version of this paper and include the following:

- KEY RESOURCES TABLE
- CONTACT FOR REAGENT AND RESOURCE SHARING
- EXPERIMENTAL MODEL AND SUBJECT DETAILS
- METHOD DETAILS
  - Flow cytometry
  - APC/C affinity purification
  - Mass spectrometry
  - *In vitro* APC/C activity assay
  - CRISPR-based genome engineering
  - Live cell microscopy
  - Measurement of SAC response by live cell imaging
  - RNAi
  - Genomic analysis of APC16 locus
  - Drug treatments
  - Plasmids and transfections
- QUANTIFICATION AND STATISTICAL ANALYSIS
  - Image analysis
  - Mass spectrometry data analysis

SUPPLEMENTAL INFORMATION

Supplemental Information includes five figures and three tables and can be found with this article online at https://doi.org/10.1016/j.celrep.2018.10.104.

ACKNOWLEDGMENTS

We thank the members of our groups for help and discussions. We thank Rajat Gupta for sharing plasmids and Elina Maskey for providing excellent technical assistance. C.C. is supported by a Hallas Møller Investigator Fellowship from the Novo Nordisk Foundation (NNF14OC0008541). This project has received funding from the European Research Council (ERC) under the European Union's Horizon 2020 Research and Innovation Program (grant agreement 648039). We thank the Mass Spectrometry Platform, the Big Data Management Platform, and the Protein Imaging Platform for excellent assistance. We thank Gelo dela Cruz from the Flow Cytometry Core Facility at the NNF Center for Stem Cell Biology (DanStem) for technical assistance with flow cytometry. The Center for Chromosome Stability is supported by the Danish National Research Foundation. The Novo Nordisk Foundation Center for Protein Research is supported financially by the Novo Nordisk Foundation (grant agreement NNF14CC0001).

AUTHOR CONTRIBUTIONS

T.W. and M. Budzowska designed, performed, and analyzed the experiments unless stated otherwise. S.H. and O.S. performed and analyzed the *in vitro* ubiquitylation assays. S.E. and M. Barisic performed and analyzed the live-cell imaging experiments shown in Figures 5E, S3B, S5D, and S5E. G.K. helped with analysis of the live-cell imaging data. T.W., M. Budzowska, and

Figure 5. Analysis of Mitosis in ΔAPC7ΔMAD2 Cells

(A) Representative images of mitosis observed for the indicated cell lines expressing H2B-mCerulean3. The frames scored as chromosome condensation and anaphase onset are indicated by the first and second arrow, respectively. Note that no anaphase onset was scored for cells that failed to form a metaphase plate under the MAD2 RNAi condition. The scale bars indicate 10 μm. Time points (in minutes) are specified in the gray bar below the images.

(B) Quantification of timing from chromatin condensation to anaphase onset from the experiment shown in (A). Cells with identical timing are illustrated as a scaled box (as described in Figure 3A). Mean timing and SD are stated on top, and median timing is drawn as a black line. Results of two independent experiments are shown, and the number of analyzed cells is stated as n. A two-tailed t test was performed to calculate significance.

(C) Table summarizing observed chromosome segregation errors in the indicated cell lines and conditions.

(D) FACS profiles of ΔMAD2ΔAPC7 and ΔMAD2-GFPΔAPC16 cells treated with reversine. Cellular DNA from wild-type, ΔAPC7ΔMAD2, and ΔMAD2-GFPΔAPC16 cells, with or without 48 hr of 0.5 μM reversine, was stained with propidium iodide and analyzed by FACS. The tables in the insets show the percentage of cells with the respective (2N–4N and >4N) DNA content.

(E) Timing from nuclear import of cyclin B1 to cyclin B1 degradation at the centrosome in the indicated cell lines and conditions. Reversine was added 2 hr before live-cell imaging, and a minimum of 20 cells were analyzed per condition. A two-tailed t test was performed to calculate significance. Error bars depict SD of the measured timings.

See also Figure S5.

C.C. wrote the manuscript. C.C. supervised the research. All authors read and commented on the manuscript.

DECLARATION OF INTERESTS

The authors declare no competing interests.

Received: December 1, 2017
Revised: September 7, 2018
Accepted: October 29, 2018
Published: November 27, 2018

REFERENCES

Alfieri, C., Chang, L., Zhang, Z., Yang, J., Maslen, S., Skehel, M., and Barford, D. (2016). Molecular basis of APC/C regulation by the spindle assembly checkpoint. Nature *536*, 431–436.

Buffin, E., Emre, D., and Karess, R.E. (2007). Flies without a spindle checkpoint. Nat. Cell Biol. *9*, 565–572.

Burds, A.A., Lutum, A.S., and Sorger, P.K. (2005). Generating chromosome instability through the simultaneous deletion of Mad2 and p53. Proc. Natl. Acad. Sci. USA *102*, 11296–11301.

Chang, L., Zhang, Z., Yang, J., McLaughlin, S.H., and Barford, D. (2015). Atomic structure of the APC/C and its mechanism of protein ubiquitination. Nature *522*, 450–454.

Cong, L., Ran, F.A., Cox, D., Lin, S., Barretto, R., Habib, N., Hsu, P.D., Wu, X., Jiang, W., Marraffini, L.A., et al. (2013). Multiplex genome engineering using CRISPR/Cas systems. Science *339*, 819–823.

Cox, J., and Mann, M. (2008). MaxQuant enables high peptide identification rates, individualized p.p.b.-range mass accuracies and proteome-wide protein quantification. Nat. Biotechnol *26*, 1367–1372.

Cox, J., Neuhauser, N., Michalski, A., Scheltema, R.A., Olsen, J.V., and Mann, M. (2011). Andromeda: a peptide search engine integrated into the MaxQuant environment. J. Proteome Res *10*, 1794–1805.

Dobles, M., Liberal, V., Scott, M.L., Benezra, R., and Sorger, P.K. (2000). Chromosome missegregation and apoptosis in mice lacking the mitotic checkpoint protein Mad2. Cell *101*, 635–645.

Elias, J.E., and Gygi, S.P. (2007). Target-decoy search strategy for increased confidence in large-scale protein identifications by mass spectrometry. Nat. Methods *4*, 207–214.

Foijer, F., DiTommaso, T., Donati, G., Hautaviita, K., Xie, S.Z., Heath, E., Smyth, I., Watt, F.M., Sorger, P.K., and Bradley, A. (2013). Spindle checkpoint deficiency is tolerated by murine epidermal cells but not hair follicle stem cells. Proc. Natl. Acad. Sci. USA *110*, 2928–2933.

Foijer, F., Albacker, L.A., Bakker, B., Spierings, D.C., Yue, Y., Xie, S.Z., Davis, S., Lutum-Jehle, A., Takemoto, D., Hare, B., et al. (2017). Deletion of the *MAD2L1* spindle assembly checkpoint gene is tolerated in mouse models of acute T-cell lymphoma and hepatocellular carcinoma. eLife *6*, e20873.

Funk, L.C., Zasadil, L.M., and Weaver, B.A. (2016). Living in CIN: Mitotic Infidelity and Its Consequences for Tumor Promotion and Suppression. Dev. Cell *39*, 638–652.

Garnett, M.J., Mansfeld, J., Godwin, C., Matsusaka, T., Wu, J., Russell, P., Pines, J., and Venkitaraman, A.R. (2009). UBE2S elongates ubiquitin chains on APC/C substrates to promote mitotic exit. Nat. Cell Biol. *11*, 1363–1369.

Hellmuth, S., Böttger, F., Pan, C., Mann, M., and Stemmann, O. (2014). PP2A delays APC/C-dependent degradation of separase-associated but not free securin. EMBO J. *33*, 1134–1147.

Hellmuth, S., Rata, S., Brown, A., Heidmann, S., Novak, B., and Stemmann, O. (2015). Human chromosome segregation involves multi-layered regulation of separase by the peptidyl-prolyl-isomerase Pin1. Mol. Cell *58*, 495–506.

Hershko, A., Ganoth, D., Sudakin, V., Dahan, A., Cohen, L.H., Luca, F.C., Ruderman, J.V., and Eytan, E. (1994). Components of a system that ligates cyclin to ubiquitin and their regulation by the protein kinase cdc2. J. Biol. Chem. *269*, 4940–4946.

Herzog, S., Nagarkar Jaiswal, S., Urban, E., Riemer, A., Fischer, S., and Heidmann, S.K. (2013). Functional dissection of the Drosophila melanogaster condensin subunit Cap-G reveals its exclusive association with condensin I. PLoS Genet 9, e1003463.

Hiruma, Y., Sacristan, C., Pachis, S.T., Adamopoulos, A., Kuijt, T., Ubbink, M., von Castelmur, E., Perrakis, A., and Kops, G.J. (2015). CELL DIVISION CYCLE. Competition between MPS1 and microtubules at kinetochores regulates spindle checkpoint signaling. Science *348*, 1264–1267.

Jensen, O.N., Wilm, M., Shevchenko, A., and Mann, M. (1999). Sample preparation methods for mass spectrometric peptide mapping directly from 2-DE gels. Methods Mol. Biol. *112*, 513–530.

Ji, Z., Gao, H., and Yu, H. (2015). CELL DIVISION CYCLE. Kinetochore attachment sensed by competitive Mps1 and microtubule binding to Ndc80C. Science *348*, 1260–1264.

Kochupurakkal, B.S., and Iglehart, J.D. (2013). Nourseothricin N-acetyl transferase: a positive selection marker for mammalian cells. PLoS One *8*, e68509.

Kops, G.J.P.L., Weaver, B.A.A., and Cleveland, D.W. (2005). On the road to cancer: aneuploidy and the mitotic checkpoint. Nat. Rev. Cancer *5*, 773–785.

Kops, G.J.P.L., van der Voet, M., Manak, M.S., van Osch, M.H.J., Naini, S.M., Brear, A., McLeod, I.X., Hentschel, D.M., Yates, J.R., 3rd, van den Heuvel, S., and Shah, J.V. (2010). APC16 is a conserved subunit of the anaphase-promoting complex/cyclosome. J. Cell Sci. *123*, 1623–1633.

Liu, X., and Winey, M. (2012). The MPS1 family of protein kinases. Annu. Rev. Biochem. *81*, 561–585.

London, N., and Biggins, S. (2014). Signalling dynamics in the spindle checkpoint response. Nat. Rev. Mol. Cell Biol. *15*, 736–747.

Magnuson, T., and Epstein, C.J. (1984). Oligosyndactyly: a lethal mutation in the mouse that results in mitotic arrest very early in development. Cell *38*, 823–833.

Markwardt, M.L., Kremers, G.-J., Kraft, C.A., Ray, K., Cranfill, P.J.C., Wilson, K.A., Day, R.N., Wachter, R.M., Davidson, M.W., and Rizzo, M.A. (2011). An improved cerulean fluorescent protein with enhanced brightness and reduced reversible photoswitching. PLoS One *6*, e17896.

Meraldi, P., Draviam, V.M., and Sorger, P.K. (2004). Timing and checkpoints in the regulation of mitotic progression. Dev. Cell *7*, 45–60.

Meyer, H.-J., and Rape, M. (2014). Enhanced protein degradation by branched ubiquitin chains. Cell *157*, 910–921.

Michel, L.S., Liberal, V., Chatterjee, A., Kirchwegger, R., Pasche, B., Gerald, W., Dobles, M., Sorger, P.K., Murty, V.V., and Benezra, R. (2001). MAD2 haplo-insufficiency causes premature anaphase and chromosome instability in mammalian cells. Nature *409*, 355–359.

Michel, L., Diaz-Rodriguez, E., Narayan, G., Hernando, E., Murty, V.V.V.S., and Benezra, R. (2004). Complete loss of the tumor suppressor MAD2 causes premature cyclin B degradation and mitotic failure in human somatic cells. Proc. Natl. Acad. Sci. USA *101*, 4459–4464.

Min, M., Mevissen, T.E.T., De Luca, M., Komander, D., and Lindon, C. (2015). Efficient APC/C substrate degradation in cells undergoing mitotic exit depends on K11 ubiquitin linkages. Mol. Biol. Cell *26*, 4325–4332.

Ong, S.-E., Blagoev, B., Kratchmarova, I., Kristensen, D.B., Steen, H., Pandey, A., and Mann, M. (2002). Stable isotope labeling by amino acids in cell culture, SILAC, as a simple and accurate approach to expression proteomics. Mol. Cell Proteomics *1*, 376–386.

Pál, M., Nagy, O., Ménesi, D., Udvardy, A., and Deák, P. (2007). Structurally related TPR subunits contribute differently to the function of the anaphase-promoting complex in Drosophila melanogaster. J. Cell Sci. *120*, 3238–3248.

Pines, J. (2011). Cubism and the cell cycle: the many faces of the APC/C. Nat. Rev. Mol. Cell Biol. *12*, 427–438.

Primorac, I., and Musacchio, A. (2013). Panta rhei: the APC/C at steady state. J. Cell Biol. *201*, 177–189.

Raaijmakers, J.A., van Heesbeen, R.G.H.P., Blomen, V.A., Janssen, L.M.E., van Diemen, F., Brummelkamp, T.R., and Medema, R.H. (2018). BUB1 Is Essential for the Viability of Human Cells in which the Spindle Assembly Checkpoint Is Compromised. Cell Rep. *22*, 1424–1438.

Shakes, D.C., Allen, A.K., Albert, K.M., and Golden, A. (2011). emb-1 encodes the APC16 subunit of the Caenorhabditis elegans anaphase-promoting complex. Genetics *189*, 549–560.

Sivakumar, S., and Gorbsky, G.J. (2015). Spatiotemporal regulation of the anaphase-promoting complex in mitosis. Nat. Rev. Mol. Cell Biol. *16*, 82–94.

Sudakin, V., Chan, G.K., and Yen, T.J. (2001). Checkpoint inhibition of the APC/C in HeLa cells is mediated by a complex of BUBR1, BUB3, CDC20, and MAD2. J. Cell Biol. *154*, 925–936.

Thornton, B.R., and Toczyski, D.P. (2003). Securin and B-cyclin/CDK are the only essential targets of the APC. Nat. Cell Biol. *5*, 1090–1094.

Townsley, F.M., Aristarkhov, A., Beck, S., Hershko, A., and Ruderman, J.V. (1997). Dominant-negative cyclin-selective ubiquitin carrier protein E2-C/UbcH10 blocks cells in metaphase. Proc. Natl. Acad. Sci. USA *94*, 2362–2367.

Wild, T., Larsen, M.S.Y., Narita, T., Schou, J., Nilsson, J., and Choudhary, C. (2016). The Spindle Assembly Checkpoint Is Not Essential for Viability of Human Cells with Genetically Lowered APC/C Activity. Cell Rep. *14*, 1829–1840.

Williamson, A., Wickliffe, K.E., Mellone, B.G., Song, L., Karpen, G.H., and Rape, M. (2009). Identification of a physiological E2 module for the human anaphase-promoting complex. Proc. Natl. Acad. Sci. USA *106*, 18213–18218.

Wirth, K.G., Ricci, R., Giménez-Abián, J.F., Taghybeeglu, S., Kudo, N.R., Jochum, W., Vasseur-Cognet, M., and Nasmyth, K. (2004). Loss of the anaphase-promoting complex in quiescent cells causes unscheduled hepatocyte proliferation. Genes Dev. *18*, 88–98.

Wu, T., Merbl, Y., Huo, Y., Gallop, J.L., Tzur, A., and Kirschner, M.W. (2010). UBE2S drives elongation of K11-linked ubiquitin chains by the anaphase-promoting complex. Proc. Natl. Acad. Sci. USA *107*, 1355–1360.

Yamaguchi, M., Yu, S., Qiao, R., Weissmann, F., Miller, D.J., VanderLinden, R., Brown, N.G., Frye, J.J., Peters, J.-M., and Schulman, B.A. (2015). Structure of an APC3-APC16 complex: insights into assembly of the anaphase-promoting complex/cyclosome. J. Mol. Biol. *427*, 1748–1764.

Yamaguchi, M., VanderLinden, R., Weissmann, F., Qiao, R., Dube, P., Brown, N.G., Haselbach, D., Zhang, W., Sidhu, S.S., Peters, J.-M., et al. (2016). Cryo-EM of Mitotic Checkpoint Complex-Bound APC/C Reveals Reciprocal and Conformational Regulation of Ubiquitin Ligation. Mol. Cell *63*, 593–607.

Yu, H., King, R.W., Peters, J.M., and Kirschner, M.W. (1996). Identification of a novel ubiquitin-conjugating enzyme involved in mitotic cyclin degradation. Curr. Biol. *6*, 455–466.

STAR★METHODS

KEY RESOURCES TABLE

REAGENT or RESOURCE	SOURCE	IDENTIFIER
Antibodies		
Anti-Mad2 Antibody, clone AS55-A12	EMD Millipore	Cat# MABE866
Anti-APC1 (D1E9D) Rabbit mAb	Cell Signaling Technology	Cat# 13329
Anti-APC2 Antibody	Cell Signaling Technology	Cat# 12301
Anti-APC7 Antibody	Bethyl laboratories	Cat# A302-551A; RRID: AB_1998911
Anti-APC8 (D5O2D) Rabbit mAb	Cell Signaling Technology	Cat# 15100
Anti-APC11 (D1E7Q) Rabbit mAb	Cell Signaling Technology	Cat# 14090
Anti-GAPDH Antibody	Merck Millipore	Cat# ABS16; RRID: AB_10806772
Anti-GFP (D5.1) XP Rabbit mAb	Cell Signaling Technology	Cat# 2956; RRID: AB_1196615
Anti-cyclin B1 Clone GNS-1	BD PharMingen	Cat# 554176; RRID: AB_395287
Anti-cyclin B1 Antibody, clone GNS3 (8A5D12)	Merck Millipore	Cat# 05-373; RRID: AB_309701
Anti- GRB2 Clone 81/GRB2	BD Transduction Laboratories	Cat# 610111; RRID: AB_397517
Anti-BubR1 Antibody	Bethyl laboratories	Cat# A300-995A; RRID: AB_2066087
Anti-Securin mAb Clone DCS-280	MBL	Cat# K0090-3; RRID: AB_592493
Anti-phospho-Histone H3 (Ser10) Antibody	Merck Millipore	Cat# 06-570; RRID: AB_310177
Anti-DNA-topoisomerase II α antibody (1C5)	Enzo Life Sciences	Cat# ADI-KAM-CC210-E; RRID: AB_2039650
Anti-mCherry/RFP antibody	Herzog et al., 2013	N/A
Anti-Cdc20 (clone E-7) antibody	Santa Cruz Biotechnology	Cat# sc-13162; RRID: AB_628089
Anti-APC3 antibody	gift from T.U. Mayer	N/A
alpha-tubulin (clone 12G10) antibody	DSHB	RRID: AB_1157911
Bacterial and Virus Strains		
DH5α E.Coli	This laboratory	N/A
Chemicals, Peptides, and Recombinant Proteins		
Nocodazole	Sigma Aldrich	Cat# M1404
reversine	MedChemexpress	Cat# HY-14711
ZM 447439	Cayman Chemicals	Cat# 13601
RPF-Trap beads	ChromoTek	RFP-Trap®_MA
Trypsin, Proteomics Grade	Sigma Aldrich	Cat# T6567
RNase A	Thermo Fisher Scientific	Cat# EN0531
Propidium Iodide	Themo Fisher Scientific	Cat# P3566
Hoechst 33342	Life Technologies	Cat# H3570
TurboFect Transfection Reagent	Thermo Fisher Scientific	Cat# R0531
Lipofectamine RNAiMAX Transfection Reagent	Thermo Fisher Scientific	Cat# 13778150
Oligonucleotides		
Silencer Select Negative Control #1 siRNA	Thermo Fisher Scientific	Cat# s813
Silencer Select MAD2 siRNA	Thermo Fisher Scientific	Cat# s8392
Silencer Select BUBR1 siRNA	Thermo Fisher Scientific	Cat# s259
See Table S2 for a list of oligonucleotide sequences	This study	N/A
Experimental Models: Cell Lines		
See Table S3 for a list of generated cell lines	This study	N/A
Software and Algorithms		
MaxQuant version 1.5.2.8	Cox and Mann, 2008	N/A
FlowJo 10.2	FlowJo LLC, Ashland, Oregon, USA	N/A
Imaris Image Analysis Software	Bitplane	N/A

CONTACT FOR REAGENT AND RESOURCE SHARING

Further information and requests for resources and reagents should be directed to and will be fulfilled by the Lead Contact, Chunaram Choudhary (chuna.choudhary@cpr.ku.dk).

EXPERIMENTAL MODEL AND SUBJECT DETAILS

HCT116 cells (American Type Culture Collection) were cultured at 37°C and 5% CO_2 in Dulbecco's modified Eagle's medium (Invitrogen) supplemented with 10% fetal bovine serum and penicillin/streptomycin. Cells were frequently tested for mycoplasma contamination.

METHOD DETAILS

Flow cytometry

To analyze the cellular DNA content, exponentially growing cells were harvested at ~70% confluency using trypsin. Cells were pelleted, resuspended in 500 μL ice-cold PBS and then permeablized by addition of 500 μL ice-cold ethanol. Samples were incubated on ice for 45 min, pelleted by centrifugation and washed with cold PBS. The cell pellet was resuspended in a solution containing propidium iodide (PI) (10 μg/ml PI in PBS) and RNase (25 μg/ml) and incubated for 30 min at 37°C. Samples were analyzed by flow cytometry using a BD LSR Fortessa (BD Biosciences). Data were analyzed and visualized using the FlowJo software (FlowJo, LLC). The Cell Cycle function in FlowJo was used to quantify cell populations with different DNA content or, in case of perturbation experiments, gates were manually set for one sample and the defined gates then applied to all samples within the same experiment.

APC/C affinity purification

APC/C was purified from APC8-mCherry expressing cells using RFP-Trap (RFP-Trap®_MA, ChromoTek). For SILAC-based quantification of proteins (Ong et al., 2002), cells were grown in SILAC medium supplemented with natural variants of L-arginine (Arg) and L-lysine (Lys), i.e., Arg^0/Lys^0 for the light SILAC condition, or supplemented with isotope labeled variants of Arg and Lys, i.e., Arg^6/Lys^4 for the medium and Arg^{10}/Lys^8 for the heavy SILAC condition. For APC/C purification, cells were lysed in RIPA buffer (50 mM Tris-HCl pH 7.5, 150 mM NaCl, 1% Nonidet P-40, 0.1% sodium-dodecyl-sulfate, 1 mM EDTA) supplemented with protease inhibitors (Complete protease inhibitor mixture tablets, Roche Diagnostics). Lysates were incubated for 10 min on ice and cleared by centrifugation at 16,000 × g. Cleared lysates were then incubated on a rotating wheel for 1 hour at 4°C with 20 μL of RFP-Trap beads. Beads were washed 3 times with RIPA buffer and eluted with LDS sample buffer (Thermo Fisher Scientific). For SILAC experiments, equal amount of proteins, as determined by Quick Start Bradford Protein Assay (Bio-Rad), were incubated with the RFP-Trap beads. The immunoprecipitates from the different SILAC conditions were washed separately with RIPA buffer once, and then combined into one new tube and subsequently washed 3 times with RIPA buffer. Eluates were analyzed by SDS-PAGE followed by immunoblotting or mass spectrometry.

Mass spectrometry

Samples in LDS sample buffer were incubated with dithiothreitol (10 mM) for 10 min at 70°C and subsequently with chloroacetamide (5.5 mM) for 60 min at 25°C. Proteins were separated on a 4%–12% gradient SDS-PAGE, stained with colloidal Coomassie blue, and digested using in-gel digestion method (Jensen et al., 1999). In brief, gel lanes were cut into six pieces and each gel piece was further sliced into smaller pieces (~1mm). Gel pieces were destained with 50% ethanol in 25 mM ammonium bicarbonate (pH 8.0) on a rotating wheel at room temperature and dehydrated with 100% ethanol. Trypsin (in 25 mM ammonium bicarbonate pH 8.0) was added to the dry gel pieces and incubated overnight at 37°C. The trypsin digestion was stopped by addition of trifluoroacetic acid (0.5% final concentration) and peptides were extracted from the gel pieces by stepwise increase in acetonitrile concentration (to 100% final). Next, acetonitrile was removed by centrifugal evaporation and peptides were purified by a C18 reversed-phase packed Stage-Tip.

Peptide fractions eluted from Stage-Tips were analyzed on a quadrupole Orbitrap (Q-Exactive, Thermo Scientific) mass spectrometer equipped with a nanoflow HPLC system (Thermo Scientific). Peptide samples were loaded onto C18 reversed-phase columns and eluted with a linear gradient from 8 to 40% acetonitrile containing 0.5% acetic acid in 105 min gradient. The Q-Exactive was operated in the data-dependent mode automatically switching between single-mass-spectrometry (MS) and tandem-mass-spectrometry (MS/MS) acquisition. Survey full-scan MS spectra (m/z 300–1700) were acquired in the Orbitrap. The 10 most intense ions were sequentially isolated and fragmented by higher-energy C-trap dissociation (HCD). Peptides with unassigned charge states, as well as peptides with charge state less than +2 were excluded from fragmentation. Fragment spectra were acquired in the Orbitrap mass analyzer.

In vitro APC/C activity assay

The *in vitro* ubiquitylation reactions were performed with the addition of 50 μg/ml recombinant UBE2S. Unless stated otherwise, purification of the APC/C and in-vitro ubiuquitylation reactions were performed as described previously (Hellmuth et al., 2014). To purify

active APC/C, HCT116 cells were synchronized at the G1/S boundary by the treatment with 2 mM thymidine (Sigma-Aldrich) for 20 hours, released into fresh medium for 6 hours, and then exposed to 0.2 μg/ml taxol (LC-laboratories). After 12-15 hours these prometaphase cells were harvested by shake-off and released for 30 min by replating them into medium supplemented with ZM 447439 (4 μM, Cayman Chemicals), taxol (0.2 μg/ml), and cycloheximide (30 μg/ml, Sigma-Aldrich). For immunoprecipitation (IP) of the APC/C, protein A Sepharose (GE Healthcare) loaded with rabbit α-APC3 (raised against a peptide with the sequence CDADDTQLHAAESDEF) was used. To start the ubiquitylation 3 μl substrate (4.5 μg recombinant securin or 3 μl cyclinB1-CDK1 from 2,3 × 10^6 transfected cells as described below) were added to a final reaction volume of 42 μl and incubated at 37°C. After 10, 25, and 45 min, 14 μl aliquots were subjected to SDS-PAGE and immunoblotted with α-securin or -cyclin B1 antibodies.

N-terminally $FLAG_3$-Tev_2 -tagged cyclin B1 and CDK1 were co-expressed in HEK293T cells by transient transfection. After 48 h cells were harvested, lysed and subjected to immunoprecipitation using mouse anti-Flag M2-Agarose (Sigma Aldrich) as described (Hellmuth et al., 2014). Bound protein was eluted in TEV cleavage buffer by addition of TEV-protease for 30 min at 18°C (Hellmuth et al., 2015).

CRISPR-based genome engineering

Crispr/Cas9 was applied to tag or delete genes of interest. DNA oligonucleotides encoding the guide RNAs were cloned into pX330 (Cong et al., 2013) and co-transfected with donor plasmids to achieve the desired genetic modification in HCT116 cells. Donor plasmids generally contained homology arms spanning approximately 500 bp from each side of the cutting site (as designated by the guide RNAs) and a selection marker, providing resistance toward puromycin, G418, zeocin, hygromycin, blasticidin or NTC (Kochupurakkal and Iglehart, 2013). For tagging endogenous genes with mCherry, EGFP, mVenus or mCerulean3 (Markwardt et al., 2011), DNA coding the respective fluorescent proteins was fused in frame to the last exon of the gene of interest. After selection, clonal cell lines were analyzed by genomic PCR and/or immunoblotting to screen for the desired genomic modification. The following guide RNAs (including PAM motif shown in bold) were used (when two guide RNAs are listed both were used simultaneously):

For gene tagging:

APC8: **cca**tagttggctactctcaagcc
MAD2: aacacaatcactaaattgca**cgg** and tgaccttttccagcagtgag**tgg**
Cyclin B1: tgtaacttgtaaacttgagt**tgg**
H2B: **cca**cgcatgttttcaataaatga and aatcatttcattcaaaaggg**ggg**
For gene deletions:
dAPC7: tgcgggacatggcggccgcg**ggg**
dAPC7intron: agcgcgactgtcacatcgct**agg** and agctcagggacccagcctcc**tgg**
dAPC16: gcccttttcacctaccccaa**agg**
dMAD2: **ccc**tgcgcgggagcgccgaaatc
See Table S3 for a list of cell lines generated in this study.

Live cell microscopy

Cells were grown in 24 well plates suited for microscopy (Imaging Plate CG, zell-kontakt). For live cell imaging, cells were seeded into FluoroBrite DMEM medium (Thermo Fisher Scientific) supplemented with 10% FBS and 4 mM L-glutamine. Live cell imaging was performed on an Olympus ScanR microscope using a 20 x objective. Images were taken continuously every 3 min acquiring 6 z stacks of 1.5 μm (H2B-mCerulean3 expressing cell lines) or every 5 min acquiring 4 z stacks of 2 μm (cyclin B1-mCerulean3 and H2B-mVenus expressing cell lines). For cyclin B1-mVenus expressing cells, time-lapse imaging was performed in a heated incubation chamber (37°C) with controlled humidity and CO2 supply (5%), using a Plan-Apochromat 63x/1.4NA oil objective with differential interference contrast, mounted on an inverted Zeiss Axio Observer Z1 microscope (Marianas Imaging Workstation from Intelligent Imaging and Innovations Inc. (3i), Denver, CO, USA), equipped with a CSU-X1 spinning-disk confocal head (Yokogawa Corporation of America) and four laser lines (405 nm, 488 nm, 561 nm and 640 nm). Images were detected using an iXon Ultra 888 EM-CCD camera (Andor Technology). Fifteen 1 μm-separated z-planes were collected every 2 minutes during 1-2 hours. Reversine (1 uM) was added to the cells 2 hours prior to filming.

Measurement of SAC response by live cell imaging

Cells were grown in 35 mm glass bottom dishes (MatTek Corporation) in DMEM supplemented with 10% Fetal Bovine Serum (FBS). To detect SAC activity, cells were treated with 100 nM nocodazole (Sigma) prior to imaging. Time-lapse imaging was performed in a heated incubation chamber (37°C) with controlled humidity and CO2 supply (5%), using a Plan-Apochromat 40x/1.4NA oil objective with differential interference contrast, mounted on an inverted Zeiss Axio Observer Z1 microscope (Marianas Imaging Workstation from Intelligent Imaging and Innovations Inc. (3i), Denver, CO, USA), equipped with a X-Cite 110 LED white light source. Images were detected using ORCA-Flash4.0 v2 sCMOS camera (Hamamatsu). Five 2.5 μm-separated z-planes were collected every 10 minutes during 16 hours.

The time from NEB to anaphase was measured from the frame where chromosomes started to condense, detected with H2B-mVenus, to the frame where chromosomes started to separate, detected with brightfield and H2B-mVenus.

RNAi

Cells were seeded into a 24-well plate and transfected with siRNAs 24 hours later using the transfection reagent RNAiMAX (Lipofectamine®) according to the manufacture's guidelines. For MAD2 depletion, the following siRNA was used: CGCCUUCGUUCAUUUACUAtt (Silencer Select, Thermo Fisher Scientific). Silencer Select Negative Control #1 siRNA (s813, Thermo Fisher Scientific) was used as a negative control. The final concentration of siRNAs was 5 nM. 24 hours after siRNA transfection, cells were trypsinized and reseeded into 24-well plates suitable for microscopy (Imaging Plate CG, zell-kontakt). 72 hours later (96 hours after siRNA transfection), Hoechst (Hoechst 33342, Life Technologies, H3570) was added directly to the medium and cells imaged with an Olympus ScanR microscope using a 20 x objective. After imaging, cell lysates were prepared from the imaged 24-wells using RIPA buffer (50 mM Tris-HCl pH 7.5, 150 mM NaCl, 1% Nonidet P-40, 0.1% sodium-dodecyl-sulfate, 1 mM EDTA). Samples containing equal amounts of protein, as determined by Bradford assay (Bio-Rad), were mixed with LDS-sample buffer and analyzed by SDS-PAGE followed by immunoblotting. For live cell imaging, cells were transfected with MAD2 siRNA 32 hours prior to imaging. For BUBR1 depletion, cells were reversely transfected with control and BUBR1 siRNA (GGAUUACUGCAUUAAACGAtt, Silencer Select, Thermo Fisher Scientific) using Lipofectamine RNAiMAX (ThermoFisher). Cells were filmed 72 hours after transfection.

Genomic analysis of APC16 locus

The genomic locus of APC16 encompassing the sequence targeted by the guide RNA was analyzed with the following primer set: gAPC16fw (5'-gcgcGGTACCgttcaaaacctgactgatattttggcctgagac) and gAPC16rev (5'-gcgcCTTAAGcgtgcccagccctgactctgcctt taacctgg). KOD Xtreme Hot Start DNA Polymerase (Novagen, Toyobo) was used according to the manufactures protocol to amplify the APC16 locus. GeneRuler 1 kb DNA Ladder (Thermo Fisher Scientific) was used as a DNA marker. To sequence potential indels within the APC16 locus, the PCR product was cloned into pcDNA3.1/ZEO (+) (Thermo Fisher Scientific, Invitrogen) using the KpnI and AflII restriction sites.

Drug treatments

To assess spindle assembly checkpoint functionality, cells were treated with 100 nM or 200 nM nocodazole (Sigma Aldrich, M1404) as indicated in the figure legends. To assay for the cellular dependence on MPS1 kinase activity, cells were treated with 0.5 μM reversine (MedChemexpress, HY-14711) for the indicated times.

Plasmids and transfections

The coding sequence of APC16 was cloned NheI/BamHI into pEGFP-N2 (Clontech), and the resulting construct (pAPC16-EGFP) was verified by sequencing. For plasmid transfection, 5 μg of plasmid (pAPC16-EGFP) was incubated in DMEM medium (without any supplements) with 15 ul TurboFect (Thermo Fisher) for 20 min at room temperature and then added to 50% confluent cells in a 10 cm dish. APC/C affinity purification was performed 32 hours after transfection.

QUANTIFICATION AND STATISTICAL ANALYSIS

Image analysis

Images acquired by live cell microscopy were analyzed using the Imaris image analysis software (Bitplane). Nuclear cyclin B1 influx was visually scored as the first frame prior to mitosis in which nuclear cyclin B1-mCerulean3 intensity equalled or exceeded cytoplasmic cyclin B1-mCerulean3 intensity. Anaphase onset was scored as the first frame in which two separate chromosome masses were observed in the H2B-mVenus channel after metaphase plate formation. To obtain cyclin B1 degradation rates, z-projections (median projections) were generated in Fiji and chromatin objects (Surfaces) were generated in Imaris using the Magic Wand tool on the H2B-mVenus channel. Mean cyclin B1 -Cerulean3 intensities of the defined chromatin objects were extracted to obtain cyclin B1 degradation trajectories for individual cells. The cyclin B1 degradation rate at anaphase onset was calculated as the slope of the linear fit for the mean cyclin B1 intensities one frame before anaphase onset, at anaphase onset, and one frame after anaphase onset. For cyclin B1-mVenus expressing cells, which lacked a chromatin marker such as a fluorocently-tagged histone, localization of cyclin B1 at the mitotic spindle poles was guided by microtubules staining using 20 nM sirTubulin and 5 uM verapamil (Spirochrome) at least 2 hours prior to filming. Timing of cyclin B1 degradation was then measured by establishing time = 0 as the frame in which cyclin B1 is imported into the nucleus (prophase) and the degradation time as the frame in which cyclin B1 was not longer detectable at the spindle poles (metaphase to anaphase transition). For H2B-mCerulean3 expressing cells, mitotic timing was calculated from the onset of chromosome condensation to anaphase onset. To score chromosome segregation errors, each analyzed cell was visually scanned through the six acquired z stacks for observable chromosome segregation defects around anaphase onset.

To analyze nuclear size and shape upon MAD2 depletion, images of Hoechst stained nuclei were visually separated into two categories: normally sized and shaped nuclei (i.e., normal nuclei), and enlarged and irregularly shaped nuclei (i.e., aberrant nuclei).

Mass spectrometry data analysis

Raw MS data were analyzed by the MaxQuant software version 1.5.2.8 (Cox and Mann, 2008). Mass spectra were searched against protein sequences from the UniProt knowledge base using the Andromeda search engine (Cox et al., 2011). Spectra were searched with a mass tolerance of 6 ppm for precursor ions, 20 ppm for fragment ions, strict trypsin specificity and allowing up to two missed cleavage sites. Cysteine carbamidomethylation was searched as a fixed modification, whereas amino-terminal protein acetylation and methionine oxidation were searched as variable modifications. A false discovery rate of less than one percent was achieved using target-decoy search strategy (Elias and Gygi, 2007) and a posterior error probability filter.

Cell Reports, Volume *25*

Supplemental Information

Deletion of APC7 or APC16 Allows Proliferation of Human Cells without the Spindle Assembly Checkpoint

Thomas Wild, Magda Budzowska, Susanne Hellmuth, Susana Eibes, Gopal Karemore, Marin Barisic, Olaf Stemmann, and Chunaram Choudhary

Figure S1

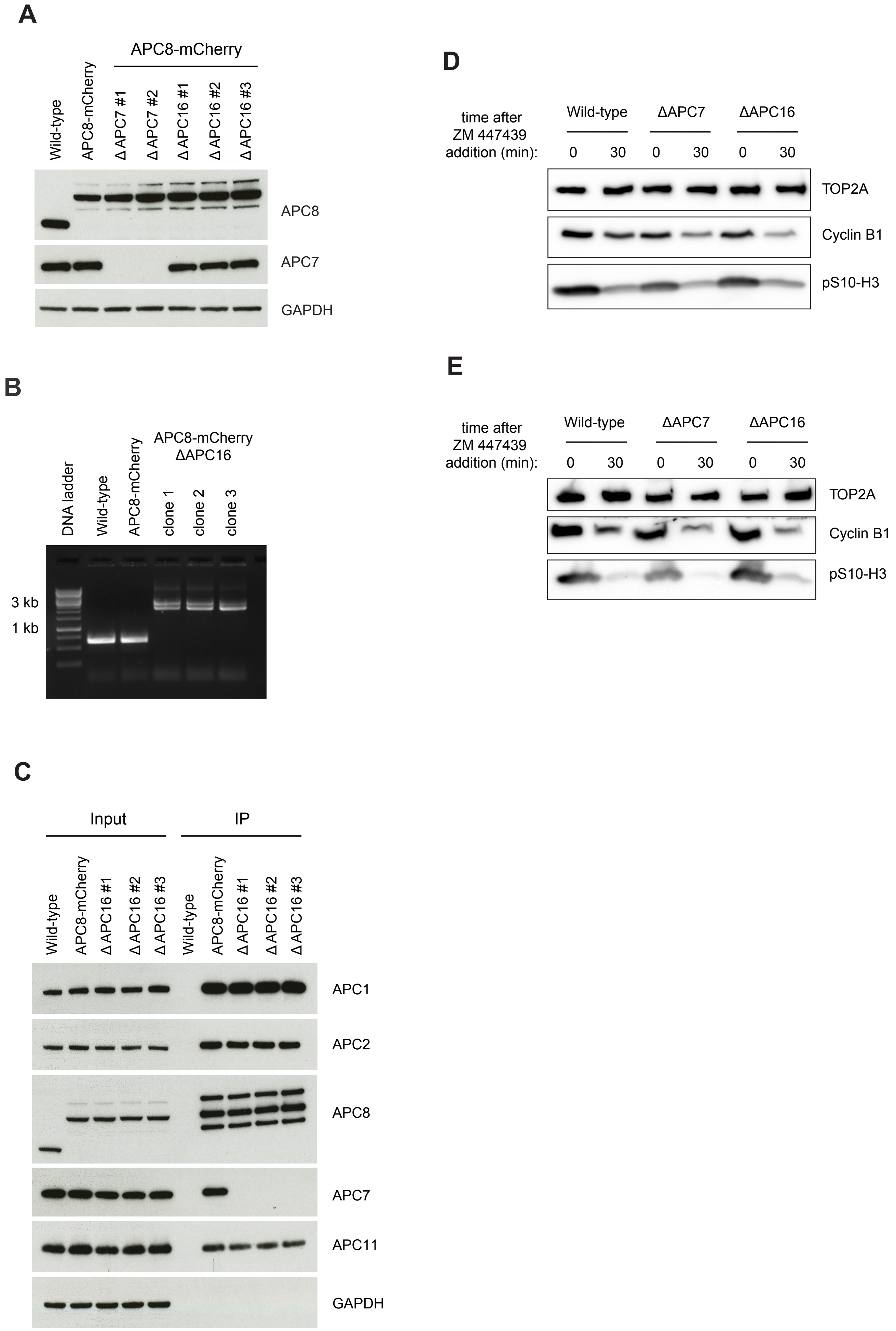
A
APC8-mCherry
Wild-type
APC8-mCherry
Δ APC7 #1
Δ APC7 #2
Δ APC16 #1
Δ APC16 #2
Δ APC16 #3
APC8
APC7
GAPDH
B
DNA ladder
Wild-type
APC8-mCherry
APC8-mCherry ΔAPC16
clone 1
clone 2
clone 3
3 kb
1 kb
C
Input
IP
Wild-type
APC8-mCherry
Δ APC16 #1
Δ APC16 #2
Δ APC16 #3
APC1
APC2
APC8
APC7
APC11
GAPDH
D
time after ZM 447439 addition (min):
Wild-type
ΔAPC7
ΔAPC16
0
30
TOP2A
Cyclin B1
pS10-H3
E
time after ZM 447439 addition (min):
Wild-type
ΔAPC7
ΔAPC16
0
30
TOP2A
Cyclin B1
pS10-H3

Figure S1. Related to Figure 1 and 2.

A) Generation of APC8-mCherry, ∆APC7 and ∆APC16 cell lines to analyse APC/C composition. ∆APC7 and ∆APC16 cells were generated in the APC8-mCherry background. Immunoblot analysis of wild-type, APC8-mCherry, APC8-mCherry ∆APC7 and APC8-mCherry ∆APC16 cell lines using the indicated antibodies. Two independent ∆APC7 and three independent ∆APC16 clonal cell lines are shown. GAPDH levels were analysed as a loading control.

B) Genomic APC16 locus from wild-type, APC8-mCherry and APC8-mCherry ∆APC16 cells analysed by PCR. The used primer pair amplifies an approximately 600 base pair (bp) product from a wild-type APC16 locus and an approximately 2300 or 2900 bp product upon genomic insertion of the deletion cassette. Note that biallelic disruption of the APC16 locus was performed using two deletion cassettes bearing different selection markers, which results in two differently sized PCR products. Three independent ∆APC16 clonal cell lines are shown.

C) Analysis of APC/C composition in ∆APC16 cells. APC/C was purified via APC8-mCherry pulldowns from APC8-mCherry and APC8-mCherry ∆APC16 cells (from three independent ∆APC16 clonal cell lines) and subsequently analyzed by immunoblotting using the indicated antibodies. Wild-type cells served as a control for unspecific binding to the affinity beads. GAPDH levels were analysed to verify equal amount of input for the different cell lines.

D) Immunoblot analysis of the indicated cell lines after the release from the prometaphase arrest used for Figure 2A. 30 min after release from taxol into ZM containing medium, cells were harvested for APC/C purification. Cyclin B1 and phospho-H3 (Ser 10) levels were assessed to compare mitotic progression, TOP2A levels were analysed as a loading control. ZM = ZM 447439.

E) Immunoblot analysis of the indicated cell lines after the release from the prometaphase arrest, used in Figure 2C, analysed as described in D).

Figure S2

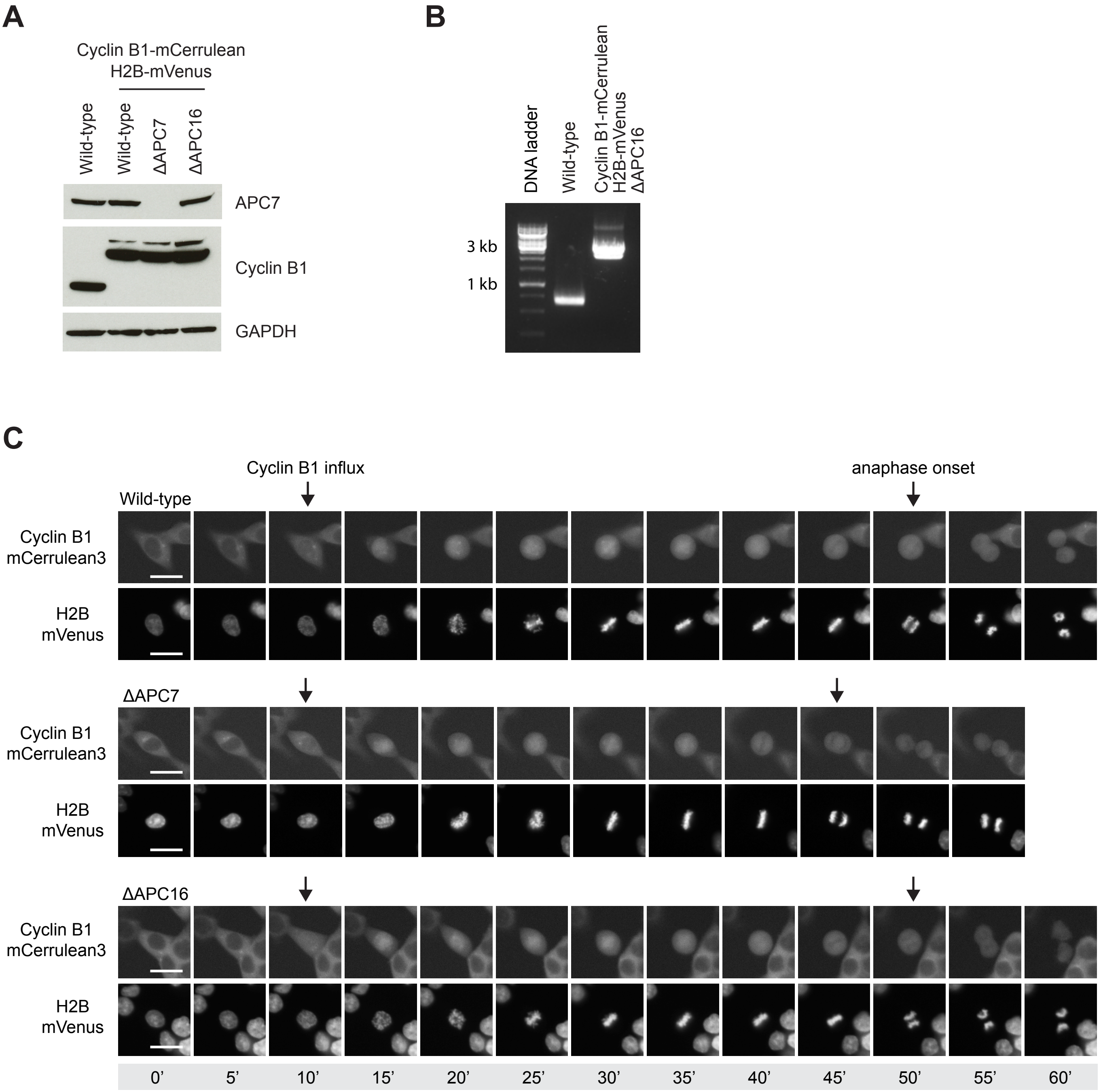
A
Cyclin B1-mCerrulean
H2B-mVenus
Wild-type
Wild-type
ΔAPC7
ΔAPC16
APC7
Cyclin B1
GAPDH
B
DNA ladder
Wild-type
Cyclin B1-mCerrulean
H2B-mVenus
ΔAPC16
3 kb
1 kb
C
Cyclin B1 influx
anaphase onset
Wild-type
Cyclin B1
mCerrulean3
H2B
mVenus
ΔAPC7
ΔAPC16
0’
5’
10’
15’
20’
25’
30’
35’
40’
45’
50’
55’
60’

Figure S2. Related to Figure 3.

A) Immunoblot analysis of the cell lines used for live cell imaging data shown in Figure 3A and 3B. Lysates from the indicated cell lines were separated by SDS-PAGE and analysed with the indicated antibodies. GAPDH levels were analysed to control for equal loading.

B) Genomic APC16 locus from wild-type and cyclin B1-mCerulean H2B-mVenus ΔAPC16 cells analysed by PCR as described in Figure S1B.

C) Representative images extracted from time-lapse imaging experiments shown in Figure 3A and Figure 3B. For the different cell lines, the cyclin B1 influx frame (cyclin B1-mCerulean3 channel) and anaphase onset frame (H2B-mVenus) are indicated as the first and second arrow, respectively. The scale bars indicate 10 μm. Time points (in minutes) are listed in the grey bar below the images.

Figure S3

A

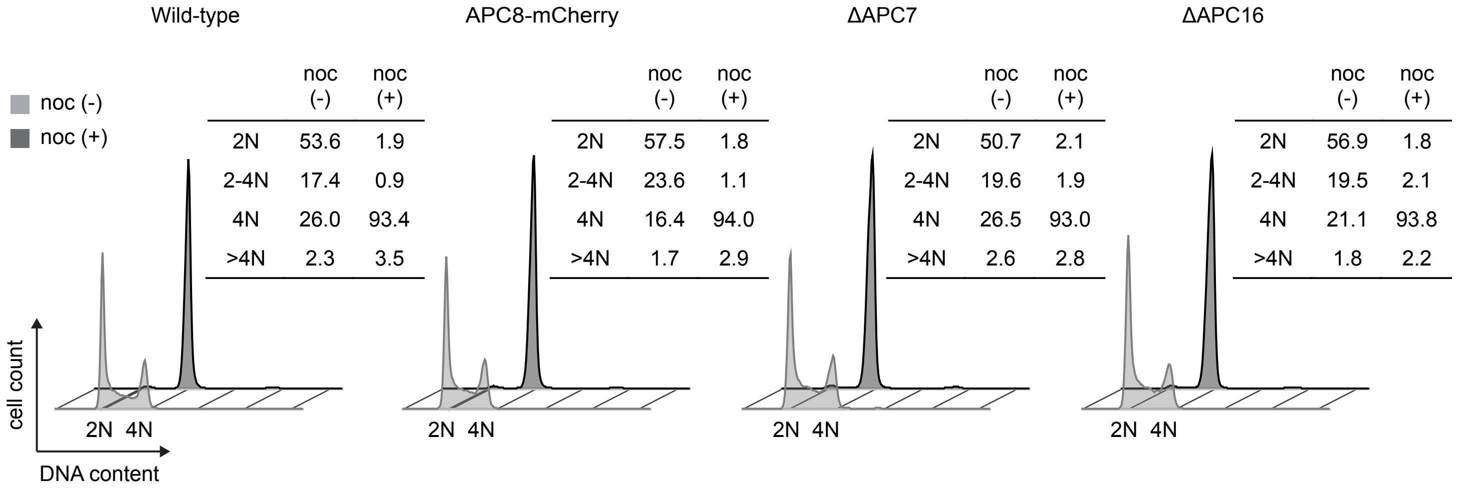

Wild-type

	noc (-)	noc (+)
2N	53.6	1.9
2-4N	17.4	0.9
4N	26.0	93.4
>4N	2.3	3.5

APC8-mCherry

	noc (-)	noc (+)
2N	57.5	1.8
2-4N	23.6	1.1
4N	16.4	94.0
>4N	1.7	2.9

ΔAPC7

	noc (-)	noc (+)
2N	50.7	2.1
2-4N	19.6	1.9
4N	26.5	93.0
>4N	2.6	2.8

ΔAPC16

	noc (-)	noc (+)
2N	56.9	1.8
2-4N	19.5	2.1
4N	21.1	93.8
>4N	1.8	2.2

B

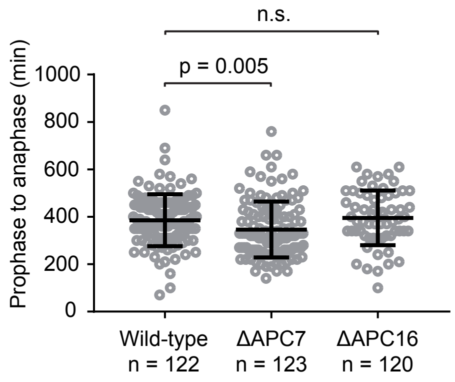

C

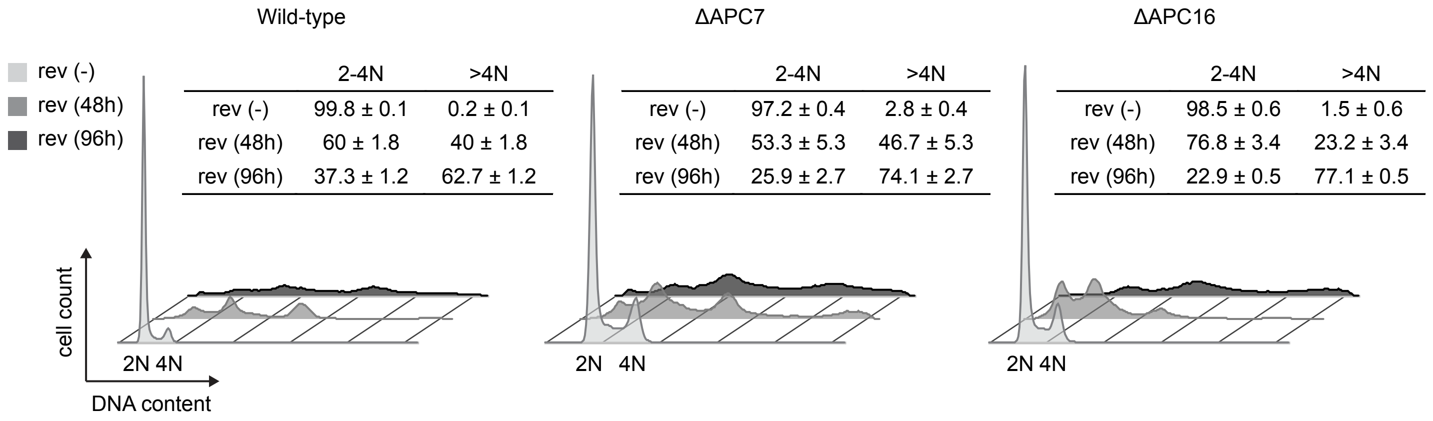

Wild-type

	2-4N	>4N
rev (-)	99.8 ± 0.1	0.2 ± 0.1
rev (48h)	60 ± 1.8	40 ± 1.8
rev (96h)	37.3 ± 1.2	62.7 ± 1.2

ΔAPC7

	2-4N	>4N
rev (-)	97.2 ± 0.4	2.8 ± 0.4
rev (48h)	53.3 ± 5.3	46.7 ± 5.3
rev (96h)	25.9 ± 2.7	74.1 ± 2.7

ΔAPC16

	2-4N	>4N
rev (-)	98.5 ± 0.6	1.5 ± 0.6
rev (48h)	76.8 ± 3.4	23.2 ± 3.4
rev (96h)	22.9 ± 0.5	77.1 ± 0.5

D

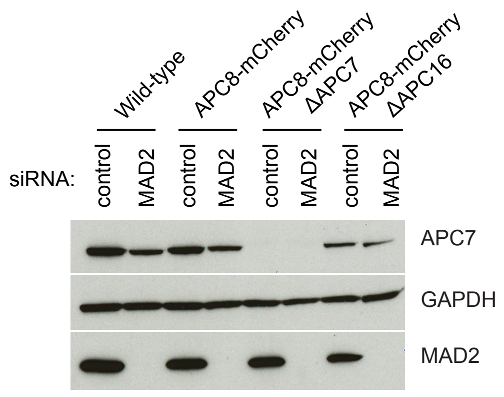

Figure S3. Related to Figure 3.

A) Analysis of cellular DNA content in wild-type, ΔAPC7 and ΔAPC16 cells upon 18 hours of nocodazole treatment. The DNA content in wild-type, ΔAPC7 and ΔAPC16 cells, grown with or without 18 hours of 200 nM nocodazole, was analyzed with propidium iodide staining and flow cytometry. The tables show the percentage of cells with the respective (2N, 2N-4N, 4N and >4N) DNA content.

B) Timing from prophase to anaphase for the indicated cell lines treated with 100 nM nocodazole. Nocodazole was added 2 hours before live cell imaging and for each cell line, the number of analysed cells is given as n. A two-tailed t-test was performed to calculate significance.

C) Analysis of cellular DNA content in wild-type, ΔAPC7 and ΔAPC16 cells upon 18 hours of nocodazole treatment. Analysis of DNA content in wild-type, ΔAPC7 and ΔAPC16 cells, grown with or without 18 hours of 200 nM nocodazole. Cells were stained with propidium iodide and analysed by flow cytometry. The tables show the percentage of cells with the respective (2N, 2N-4N, 4N and >4N) DNA content.

D) Immunoblot analysis of control and MAD2 RNAi used in Figure 3E-F. Cells imaged for Figure 3E and Figure 3F were lysed and expression of the indicated proteins was analysed by immunoblotting. A representative blot of the three independently performed experiments is shown. GAPDH levels were analysed to control for equal loading.

Figure S4

A

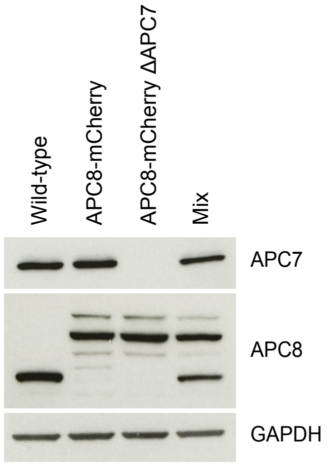

B

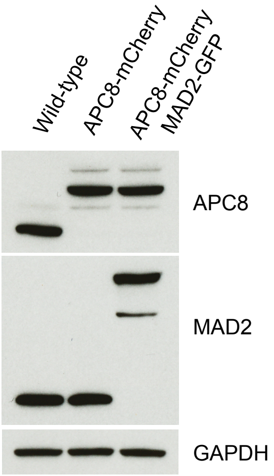

C

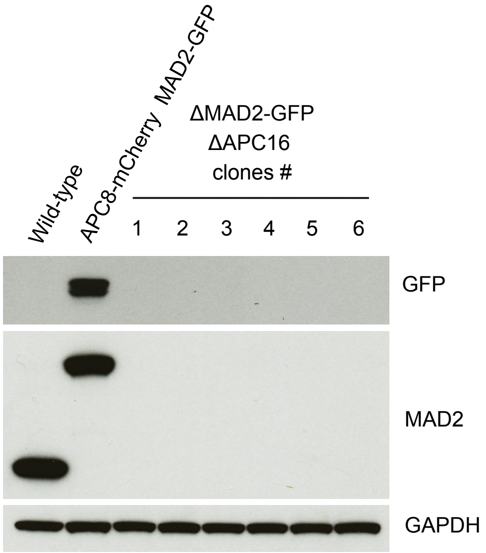

D

APC8-mCherry
ΔMAD2-GFP
ΔAPC16
clone #1

Allele 1:
ATGGCTGCTTCATCATCATCCTCCTCAGCTGGTGGGGTCAGTGGAAGTTCTGTCACTGGATCTGGTTTCAGT
GTCTCAGACCTTGCCCCACCACGGAAAGCCCTTTTCACCTACCCCAA**AGG**AGCTGGAGAGATGTTAGAAG

Allele 2:
ATGGCTGCTTCATCATCATCCTCCTCAGCTGGTGGGGTCAGTGGAAGTTCTGTCACTGGATCTGGTTTCAGT
GTCTCAGACCTTGCCCCACCACGGAAAGCCCTTTTCACCTACCCCAA**AGG**AGCTGGAGAGATGTTAGAAG

APC8mCherry
ΔMAD2-GFP
ΔAPC16
clone #2

Allele 1:
ATGGCTGCTTCATCATCATCCTCCTCAGCTGGTGGGGTCAGTGGAAGTTCTGTCACTGGATCTGGTTTCAGT
GTCTCAGACCTTGCCCCACCACGGAAAGCCCTTTTCACCTACCCCAA**AGG**AGCTGGAGAGATGTTAGAAG

Allele 2:
ATGGCTGCTTCATCATCATCCTCCTCAGCTGGTGGGGTCAGTGGAAGTTCTGTCACTGGATCTGGTTTCAGT
GTCTCAGACCTTGCCCCACCACGGAAAGCCCTTTTCACCTACCCCAA**AGG**AGCTGGAGAGATGTTAGAAG

APC8-mCherry
ΔMAD2-EGFP
ΔAPC16
clone #3

Allele 1:
ATGGCTGCTTCATCATCATCCTCCTCAGCTGGTGGGGTCAGTGGAAGTTCTGTCACTGGATCTGGTTTCAGT
GTCTCAGACCTTGCCCCACCACGGAAAGCCCTTTTCACCTACCCCAA**AGG**AGCTGGAGAGATGTTAGAAG

Allele 2:
ATGGCTGCTTCATCATCATCCTCCTCAGCTGGTGGGGTCAGTGGAAGTTCTGTCACTGGATCTGGTTTCAGTGT
CTCAGACCTTGCCCCACCACGGAAAGCCCTTTTCACCTACCCCAA**AGG**AGCTGGAGAGATGTTAGAAG

APC8-mCherry
ΔMAD2-GFP
ΔAPC16
clone #4

Allele 1:
ATGGCTGCTTCATCATCATCCTCCTCAGCTGGTGGGGTCAGTGGAAGTTCTGTCACTGGATCTGGTTTCAGTGT
CTCAGACCTTGCCCCACCACGGAAAGCCCTTTTCACCTACCCCAA**AGG**AGCTGGAGAGATGTTAGAAG

Allele 2:
ATGGCTGCTTCATCATCATCCTCCTCAGCTGGTGGGGTCAGTGGAAGTTCTGTCACTGGATCTGGTTTCAGTGT
CTCAGACCTTGCCCCACCACGGAAAGCCCTTTTCACCTACCCAGTATAATCTGCAGTCAATATTTCTGCGAAAGC
TTACTACTTCAAAGCCTTAAAAAGTGCATACTTTGGTCCAGCAAGTACCATTCCAATAATTTTTCTGAGAAAATAAT
CAAGGATACCAA**AGG**AGCTGGAGAGATGTTAGAAG

E

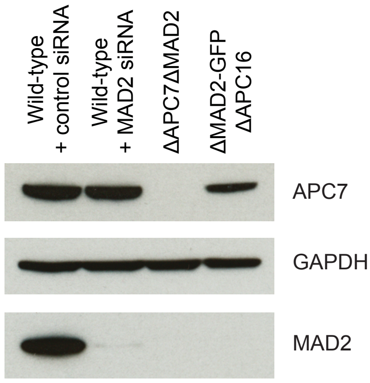

Figure S4. Related to Figure 4.

A) Immunoblot of cells used for mixing experiment outlined in Figure 4A. Equal number of wild-type, APC8-mCherry and APC8-mCherry ΔAPC7 cells were mixed and seeded together. Lysate from the individual cell lines and the resulting mixed cell population (on the day of transfection of MAD2-targeting Crispr/Cas9 plasmids) were analysed for expression of indicated proteins with immunoblotting.
B) Immunoblot analysis of the APC8-mCherry MAD2-GFP cell line along with its parental cell lines with the indicated antibodies.
C) Immunoblot analysis of MAD2 expression in the six individual clones retrieved from MAD2-GFP synthetic viability assay performed in combination with an APC16 targeting guide RNA. Immunoblot for GAPDH serves as loading control.
D) Confirmation of APC16 locus disruption in four ΔMAD2-GFPΔAPC16 clones. The genomic APC16 locus surrounding the guide RNA target site was cloned from four different ΔMAD2-GFPΔAPC16 clonal cell lines and sequenced. The table lists the obtained sequences, beginning with the start codon of APC16 and depicting the PAM sequence adjacent to the guide RNA in bold. Compared to the wild-type APC16 locus, deleted base pairs are shown in boxes, inserted base pairs are highlighted with grey background. Note that all sequenced mutations cause disruption of the APC16 reading frame.
E) Immunoblot analysis of ΔAPC7ΔMAD2, ΔMAD2-GFPΔAPC16 and MAD2 RNAi in wild-type HCT116 cells, used in Figure 4E. A representative blot of the three independently performed experiments is shown. GAPDH levels were analysed to control for equal loading.

Figure S5

A

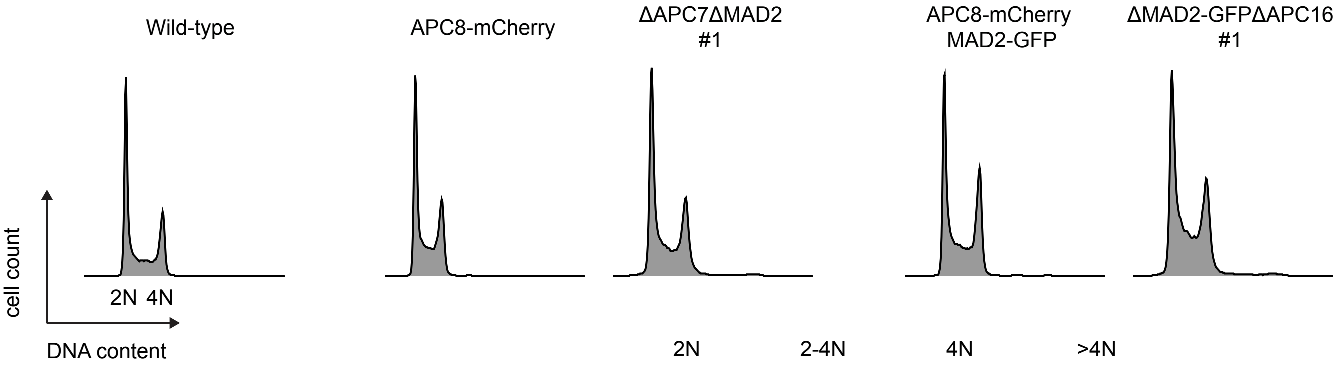

	2N	2-4N	4N	>4N
Wild-type	35 ± 2	43 ± 2	18 ± 1	2 ± 0.3
APC8-mCherry	33 ± 1	44 ± 0.1	19 ± 1	2 ± 0.4
ΔAPC7ΔMAD2 (clones 1-4)	30 ± 2	42 ± 2	18 ± 0.4	7 ± 1
APC8-mCherry MAD2-GFP	27 ± 1	49 ± 1	20 ± 1	3 ± 1
ΔMAD2-GFPΔAPC16 (clones 1-4)	26 ± 2	46 ± 1	18 ± 1	7 ± 1

B

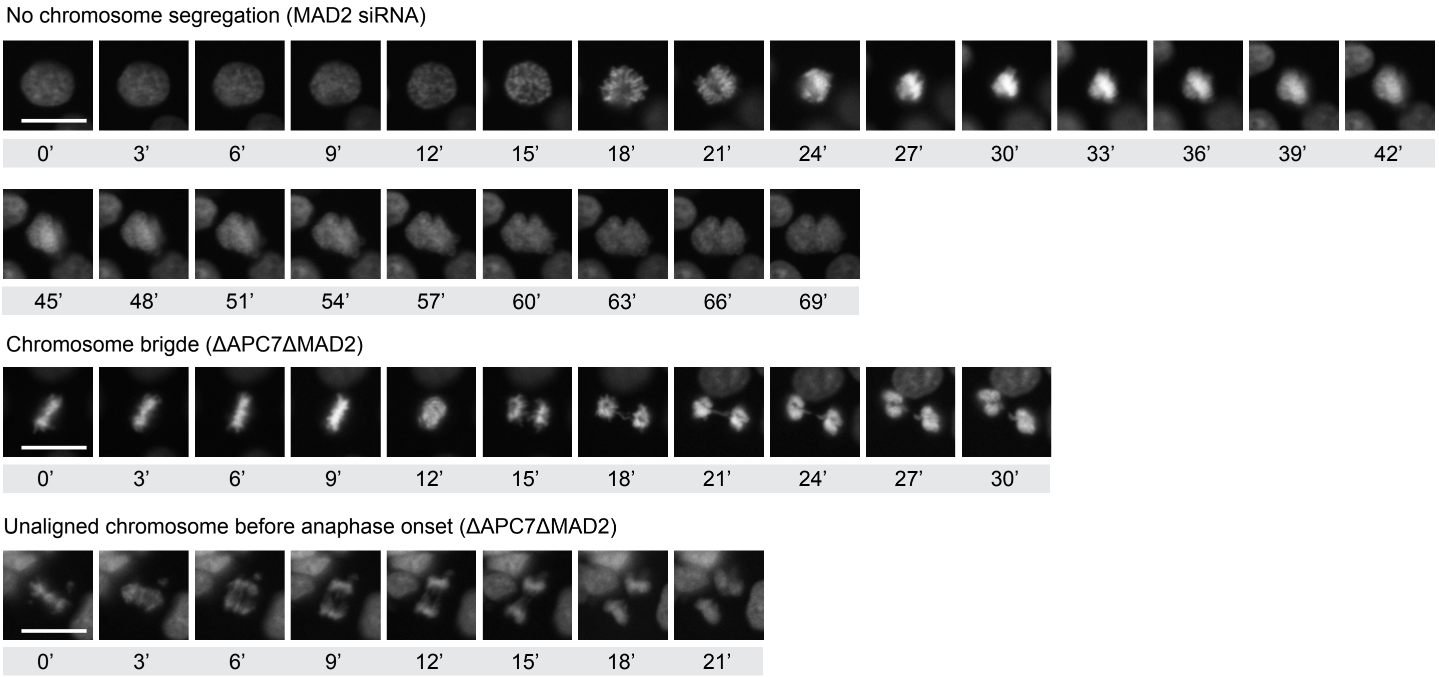

C

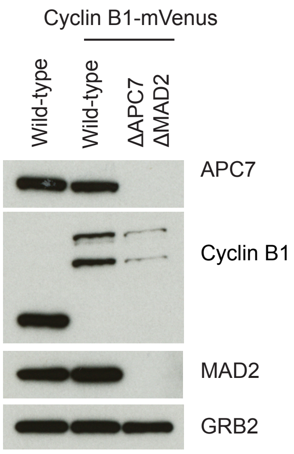

D

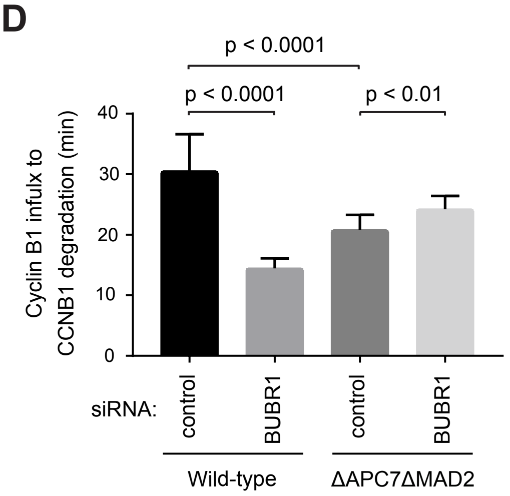

E

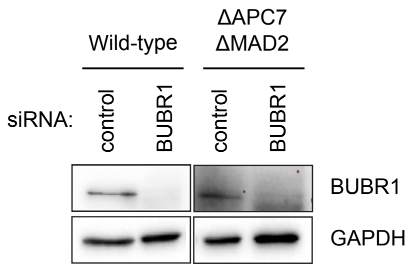

Figure S5. Related to Figure 5.

A) Analysis of DNA content in ΔAPC7ΔMAD2 and ΔMAD2-GFPΔAPC16 clonal cell lines cells. The cellular DNA from wild-type, APC8-mCherry, ΔAPC7ΔMAD2, APC8-mCherry MAD2-GFP, and ΔMAD2-GFPΔAPC16 clonal cell lines was stained with propidium iodide and analysed by FACS. The table shows the percentage of cells with the respective (2N, 2N-4N, 4N and >4N) DNA content.
B) Representative images of chromosome segregation errors summarized in Figure 5C. The scale bars indicate 10 μm. Time points (in minutes) are listed in the grey bar below the images.
C) Immunoblot analysis of the indicated cell lines, used in Figure 5E, S5D, S5E. GRB2 levels were analysed to control for equal loading.
D) Timing form nuclear cyclin B1 import to cyclin B1 degradation at centrosomes from the indicated cell lines and conditions. Live cell imaging was performed 72 hours after siRNA transfection. At least 10 cells per condition were analysed. Error bars depict the standard deviation of the measured timings.
E) Immunoblot analysis of BUBR1 depletion by RNAi in the indicated cell lines, used in D). GAPDH levels were analysed to control for equal loading.
